# Deciphering the metabolome in Alzheimer’s disease

**DOI:** 10.1186/s13024-026-00982-w

**Published:** 2026-07-31

**Authors:** Xiaoli Yang, Yong Shen

**Affiliations:** 1 Program of Bone-Brain Axis Research, Department of Orthopedics, Institute on Aging and Brain Disorders, The First Affiliated Hospital of USTC, Division of Life Sciences and Medicine, University of Science and Technology of China, Hefei, China; 2 Neurodegenerative Disorder Research Center, Hefei National Laboratory for Physical Sciences at the Microscale, Division of Life Sciences and Medicine, University of Science and Technology of China, Hefei, China; 3 Center for Excellence in Brain Science and Intelligence Technology, Chinese Academy of Sciences, Shanghai, China; 4 Anhui Province Key Laboratory of Biomedical Aging Research, University of Science and Technology of China, Hefei, China

## Abstract

Alzheimer’s disease (AD), a multifaceted neurodegenerative disease, stands as the most prevalent form of dementia and one of the leading causes of death in the elderly. An increasing body of evidence underscores the intricate link between AD and metabolic disturbances. Metabolomics research has unveiled dysregulations in various metabolites within the brain and body fluids of individuals with AD, shedding light on the disease’s metabolic underpinnings. Furthermore, notable correlations were identified between particular metabolites and AD-associated pathologies, as well as cognitive phenotypes, throughout the course of AD progression. Despite these valuable insights, a holistic understanding of the metabolic basis of AD is still scarce. This review examines recent studies investigating metabolome changes in different types of specimens from AD individuals. We presented a summary of the metabolic signatures of AD and the correlation between metabolites and AD-related traits. We then delineated the various biochemical pathways by which metabolites are involved in AD pathogenesis. We hope that this review will provide a basis for the further development of biomarkers and therapeutic strategies for metabolic disorders in AD.

**Supplementary Information:**

The online version contains supplementary material available at https://doi.org/10.1186/s13024-026-00982-w.

## Introduction

Alzheimer’s disease (AD) is a progressive neurodegenerative disorder marked by memory loss and other cognitive impairment. Many potential pathological mechanisms underlying AD have been proposed [1], and in particular, the amyloid cascade and the neurofibrillary tangles (NFTs) are widely acknowledged [2]. These occur due to the aggregation and deposition of toxic proteins, including amyloid-β (Aβ) and tau [3]. Beyond protein-related processes, small molecule disturbances have been implicated as metabolic drivers in AD pathophysiology during dynamic disease progression [4]. There is a growing body of evidence that AD individuals experience central and peripheral metabolic dysfunction, and the mechanisms underlying such events are being gradually identified [5].

Small molecules that appear as intermediates or end products in metabolic reactions are called metabolites [6], which have a molecular weight of less than 1500 Da [7]. There are different types of metabolites, including sugars, amino acids (AAs), peptides, lipids, polyamines, organic acids, and many more [8, 9]. In addition to supplying energy, structural components and materials for macromolecules synthesis, metabolites also serve as signaling molecules [10]. Given the direct reflection of physiological states, metabolomes can be used to predict individual disease outcomes [11]. Metabolomics technology, particularly in recent years, has gained attention for discovering AD biomarkers [12]. A number of altered metabolites were identified by multiple metabolomic platforms in brain tissues, cerebrospinal fluid (CSF), peripheral blood, urine, and saliva from AD individuals [13]. Furthermore, the underlying mechanisms by which metabolites play vital roles in AD pathology have also been elucidated [14–17].

Here, we review recent advances in this field by summarizing metabolomics studies conducted on various types of clinical samples across different stages of AD. We discuss the correlations between metabolites and AD pathologies, as well as their associations with cognitive function, along with the potential of metabolites to enhance the clinical diagnosis of AD. We subsequently present an overview of the molecular pathways through which metabolites contribute to AD hallmark pathologies, which may provide insights for the development of targeted metabolic treatment strategies for AD. Finally, the implications of metabolome research for advancing clinical translation and accelerating mechanistic exploration of AD are discussed.

## The disruption of metabolites throughout the brain in AD

**Fig. 1:**
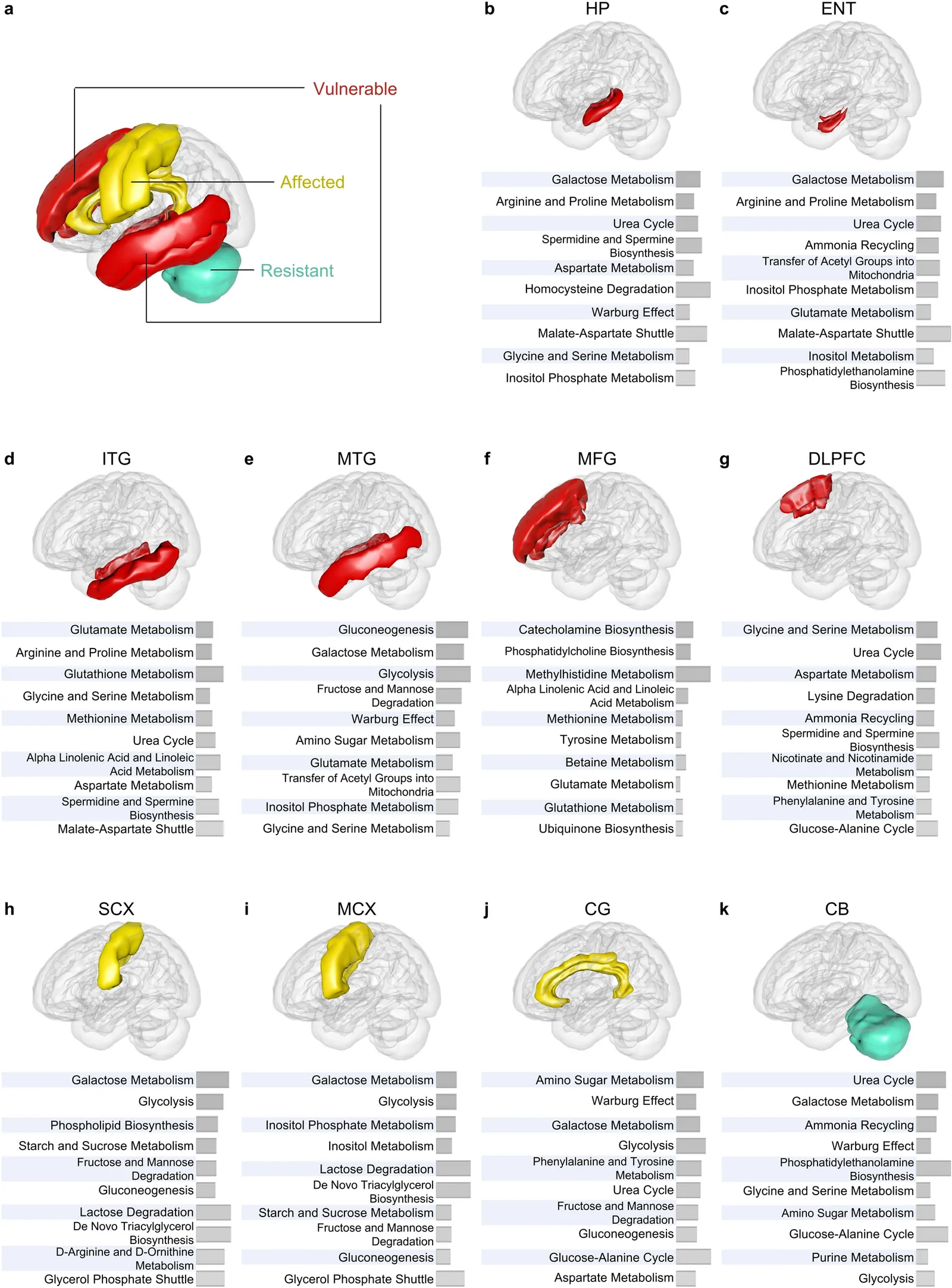
Metabolic pathways change in various brain regions of AD individuals. **a**, Brain regions that are known to be vulnerable (red), affected (yellow), and relatively spared (green) during AD progression. **b-k**, The top 10 enriched pathways of differential metabolites reported in the HP (**b**), ENT (**c**), ITG (**d**), MTG (**e**), MFG (**f**), DLPFC (**g**), SCX (**h**), MCX (**i**), CG (**j**), and CB (**k**) in AD individuals. The brain region images in this figure were generated by the Scalable Brain Atlas [39] using the Human Brainnetome Atlas template [40]. The pathway enrichment analysis of differential metabolites was performed using MetaboAnalyst 6.0 based on the metabolite sets from SMPDB. The full analysis results were listed in Supplementary Table 1

A combination of extracellular amyloid plaques and intraneuronal NFTs characterize AD brains microscopically [18]. It was not clear, however, what happens inside the brain as AD progresses in terms of metabolites [19]. For this reason, metabolomics techniques employing high-resolution mass spectrometry (MS) are applied to biochemically profile post-mortem brain tissues of subjects with AD in order to study brain metabolites [20]. The disruption of metabolites has been observed in brain regions that are vulnerable to AD pathology: hippocampus (HP) [21–25], entorhinal cortex (ENT) [21], inferior temporal gyrus (ITG) [19, 26–30], middle-temporal gyrus (MTG) [21], middle frontal gyrus (MFG) [19, 27–30], and dorsolateral prefrontal cortex (DLPFC) [31–34]; moderately affected: sensory cortex (SCX) [21], motor cortex (MCX) [21], and cingulate gyrus (CG) [21, 22]; and relatively spared: cerebellum (CB) [19, 21, 25, 28] (Table 1; Fig. 1a). Therefore, it is more likely that the impacts of AD on brain metabolism are global rather than restricted to specific regions [21]. Meanwhile, Different brain regions exhibit distinct changes in certain metabolites (Table 1), as well as differences in the enriched pathways of differential metabolites in AD (Fig. 1b-k and Supplementary Table 1). These enrichment results provide detailed insights into the metabolic characteristics of brain regions varying in both functional specialization and susceptibility to AD pathological damage, while highlighting potential therapeutic targets tailored to these regions.

**Table 1:** The disruption of metabolites in various brain regions of individuals with AD

Metabolites	Vulnerable						Affected			Resistant
	HP	ENT	ITG	MTG	MFG	DLPFC	SCX	MCX	CG	CB
**Glucose and other fuel sources related metabolites**										
Glucose	↑ [21]	↑ [21]	↑ [30]	↑ [21]	↑ [30]		↑ [21]	↑ [21]	↑ [21]	↑ [21]
Glucose-6-phosphate	↑ [21]	↑ [21]		↑ [21]			↑ [21]	↑ [21]	↑ [21]	↑ [21]
Sorbitol	↑ [21]	↑ [21]		↑ [21]		↑ [31]	↑ [21]	↑ [21]	↑ [21]	↑ [21]
Fructose	↑ [21]	↑ [21]		↑ [21]			↑ [21]	↑ [21]	↑ [21]	↑ [21]
Fructose-6-phosphate				↑ [21]					↑ [21]	
Pentonic acid A		↑ [21]		↑ [21]					↑ [21]	
Pentonic acid B	↑ [21]	↑ [21]		↑ [21]			↑ [21]	↑ [21]	↑ [21]	
Arabinose	↑ [21]			↑ [21]			↑ [21]	↑ [21]	↑ [21]	
Ribose-5-phosphate		↓ [21]		↓ [21]						
Erythronic acid	↑ [21]	↑ [21]								
Butanediol		↑ [21]					↑ [21]	↑ [21]	↑ [21]	↑ [21]
β-Hydroxybutyric acid							↑ [21]		↑ [21]	
Lactate	↑ [21]									
2-Hydroxy-3-methylbutyric acid	↑ [21]						↑ [21]		↑ [21]	
Threitol	↑ [21]	↑ [21]		↑ [21]			↑ [21]	↑ [21]	↑ [21]	↑ [21]
Xylitol	↑ [21]								↑ [21]	
Disaccharide	↑ [21]			↑ [21]					↑ [21]	
GlcNAc				↑ [21]					↑ [21]	
Myo-Inositol	↑ [22, 23]	↑ [21]				↑ [31]				
Myo-Inositol-1-phosphate	↑ [21]	↑ [21]		↑ [21]				↑ [21]	↑ [21]	↑ [21]
Glycerol	↓ [21]	↓ [21]								
Glycerol-2-phosphate	↑ [21]	↑ [21]		↑ [21]					↑ [21]	↑ [21]
Glycerol-3-phosphate	↑ [21]	↑ [21]					↑ [21]	↑ [21]	↑ [21]	↑ [21]
Glyceric acid				↑ [21]					↑ [21]	↑ [21]
Citric acid	↑ [21, 24]	↑ [21]								
Malic acid		↑ [21]		↑ [21]						
1,5-Anhydroglucitol						↑ [31, 34]				
Uridine diphosphate glucose						↑ [31]				
Ribitol						↓ [31]				
**Amino acids and derivatives**										
Proline	↓ [21]	↓ [21]								
Lysine	↓ [21]					↑ [31]				
Glycine		↓ [21]	↓ [28]	↓ [21]						
Serine	↓ [21, 24]	↓ [21]								↓ [21]
Threonine				↑ [21]		↑ [31, 33, 34]				
Cysteine	↑ [21]		↑ [29]		↑ [29]				↑ [21]	
β-Alanine				↑ [21]						
Phenylalanine	↓ [24]			↑ [21]		↑ [31, 33]			↑ [21]	
Tryptophan	↑ [21]	↑ [21]	↑ [28]	↑ [21]		↑ [31, 33]		↑ [21]	↑ [21]	
Aspartic acid	↓ [21]	↓ [21]								
NAA	↓ [22], ↑ [24]		↓ [29]	↓ [21]		↓ [32]			↓ [22]	
Ornithine	↓ [21, 25]	↓ [21]	↑ [28]			↑ [31]				↓ [21, 25]
Glutamic acid						↑ [33]			↑ [21]	
GABA	↑ [24]	↓ [21]	↑ [28], ↓ [29]	↓ [21]	↑ [28], ↓ [29]		↓ [21]			
Glutamate	↓ [23, 25]		↓ [28]			↑ [31, 34]				↑ [25]
Isoleucine	↓ [24]									
Leucine	↓ [24]					↑ [31, 34]				
Tyrosine	↓ [24]				↑ [28]	↑ [31, 33]				
Valine	↓ [24]					↑ [31]				
Glutamine						↑ [33]				↑ [25]
Aspartate			↓ [28]							
Arginine			↑ [28]			↑ [31]				
Betaine						↓ [31, 34]				
Asparagine						↑ [31]				
Allo-Threonine						↑ [31]				
Methionine						↑ [33]				
Histidine						↑ [33]				
Alanine						↑ [33]				
Argininate						↑ [34]				
NAG		↓ [21]	↓ [29]		↓ [29]	↓ [31, 34]				
SAH					↑ [29]					
SAM			↑ [29]		↑ [29]					
Glutathione			↑ [29]		↑ [29]					
Homocarnosine						↓ [31]				
NAAG						↓ [31, 34]				
2-Aminoadipate						↑ [31]				
β-Citrylglutamate						↓ [31, 34]				
N-acetylglycine						↑ [31, 34]				
4-Acetamidobutanoate						↓ [31]				
Pipecolate						↓ [31]				
Methionine sulfoxide						↑ [31, 34]				
N-Acetylglutamine						↑ [31]				
Guanidinoacetate						↑ [31, 34]				
1-Carboxyethylleucine						↑ [31]				
Symmetric dimethylarginine						↑ [31, 33]				
1-Carboxyethylphenylalanine						↑ [31, 34]				
γ-Glutamylthreonine						↑ [31, 34]				
Carboxyethyl-GABA						↑ [31, 34]				
N-Acetylmethionine sulfoxide						↑ [31]				
N-Monomethylarginine						↑ [31]				
Methionine sulfone						↑ [31]				
1-Carboxyethylvaline						↑ [31]				
1-Carboxyethyltyrosine						↑ [31]				
N-Acetyl-3-methylhistidine						↓ [31]				
α-Aminoadipic acid						↑ [33]				
2-Oxoarginine						↑ [34]				
3-Methylcytidine						↑ [34]				
Cysteinylglycine disulfide						↑ [34]				
γ-Glutamylvaline						↑ [31, 34]				
N-Acetylhistidine						↓ [34]				
Saccharopine						↑ [34]				
4-Hydroxy-nonenal-glutathione						↑ [31]				
Creatine	↑ [24]									
Putrescine	↓ [25]					↓ [31, 33]				
Spermine	↑ [25]									
Spermidine			↑ [26, 29]							
Serotonin			↓ [28]							
L-DOPA					↑ [28]					↑ [28]
Dopamine					↓ [28]					
N-Acetylputrescine						↓ [31]				
Acisoga						↑ [31]				
**Nucleosides**										
Adenine										↑ [21]
Uracil	↓ [21]	↓ [21]		↓ [21]			↓ [21]	↓ [21]	↓ [21]	↓ [21]
Adenosine-5-monophosphate		↑ [21]								
Guanosine			↓ [28]	↑ [21]	↓ [28]					
Hypoxanthine		↓ [21]		↓ [21]		↓ [31]	↓ [21]			↓ [21]
2’-Deoxycytidine						↓ [31]				
**Lipids and derivatives**										
LA			↓ [19]		↓ [19]					
OA			↓ [19]		↓ [19]					
DHA			↑ [19]		↑ [19]					↑ [19]
ARA			↓ [19]		↓ [19]					
LNA			↓ [19]		↓ [19]					
EPA			↓ [19]		↓ [19]					↓ [19]
4-Hydroxybutyric acid		↓ [21]								
15-KETE						↑ [31, 34]				
Maleate						↑ [31]				
Caprate (10:0)						↓ [31]				
Phosphocholine	↑ [24]									
LysoPC a C18:0			↓ [26]							
PC aa C40:4			↓ [26]							
PC ae C34:0			↓ [26]							
PC ae C36:0			↓ [26]							
PC ae C36:4			↑ [26]							
PC ae C40:1			↓ [26]							
PC ae C42:3			↓ [26]							
PC aa C36:0						↓ [33]				
PC ae C38:1						↓ [33]				
PC ae C42:2						↓ [33]				
PC ae C38:4						↓ [33]				
PC aa C38:3						↓ [33]				
PC ae C42:1						↓ [33]				
PC ae C36:1						↓ [33]				
GPC						↑ [31]				
GPE						↑ [31]				
1-Palmitoyl-2-arachidonoyl-GPE (16:0/20:4)						↓ [31]				
1-Stearoyl-2-oleoyl-GPE (18:0/18:1)						↓ [31]				
1-Linoleoyl-2-arachidonoyl-GPC (18:2/20:4n6)						↓ [31]				
1-Stearoyl-2-oleoyl-GPE (18:0/18:1)						↓ [31]				
1-Stearoyl-2-arachidonoyl-GPE (18:0/20:4)						↓ [31]				
1-Oleoyl-2-arachidonoyl-GPE (18:1/20:4)						↓ [31]				
1-Palmitoyl-2-alpha-linolenoyl-GPC (16:0/18:3n3)						↓ [31]				
1-oleoyl-2-docosahexaenoyl-GPC(18:1/22:6)						↓ [31]				
GPI						↓ [31]				
1,2-Dilinoleoyl-GPC (18:2/18:2)						↓ [31]				
1-Palmitoyl-2-arachidonoyl-GPI (16:0/20:4)						↓ [31]				
1-Palmitoyl-2-oleoyl-GPE (16:0/18:1)						↓ [31]				
1-Stearoyl-GPS (18:0)						↑ [31]				
1-Palmitoyl-2-dihomo-linolenoyl-GPC (16:0/20:3n3 or 6)						↓ [31]				
1,2-Dioleoyl-GPE (18:1/18:1)						↓ [31]				
1-(1-enyl-stearoyl)-2-arachidonoyl-GPE (P-18:0/20:4)						↓ [31]				
1-Palmitoyl-GPS (16:0)						↑ [34]				
1-Stearoyl-GPS (18:0)						↑ [34]				
SM C16:0			↑ [26]							
SM C16:1			↑ [26]							
SM C24:1			↑ [26]							
SM C26:1			↑ [26]							
SM (OH) C14:1			↑ [26]							
SM (OH) C22:1			↑ [26]							
SM (OH) C22:2			↑ [26]							
SM (OH) C24:1			↑ [26]							
Lanosterol					↓ [27]					
24 S-Hydroxycholesterol			↓ [27]							
5α,6α-Epoxycholesterol			↑ [27]							
5α,6β-Dihydroxycholestanol			↑ [27]							
5β,6β-Epoxycholesterol			↑ [27]							
7-Ketocholesterol			↑ [27]							
7β-Hydroxycholesterol			↑ [27]			↑ [31]				
4-Cholesten-3-one						↑ [31]				
Deoxycarnitine						↓ [31]				
Propionylcarnitine						↑ [33]				
Isobutyrylcarnitine						↑ [31]				
Glutarylcarnitine						↑ [31]				
**Others**										
Fumaric acid									↑ [21]	
Urea	↑ [21]	↑ [21]		↑ [21]			↑ [21]	↑ [21]	↑ [21]	↑ [21]
Creatinine				↑ [21]		↑ [33]				↑ [21]
Ethanolamine	↓ [21]	↓ [21]		↓ [21]			↓ [21]	↓ [21]	↓ [21]	↓ [21]
Methyl-phosphate		↓ [21]		↓ [21]						
Phosphoric acid								↓ [21]		
2-Hydroxyglutaric acid	↑ [21]	↑ [21]		↑ [21]				↑ [21]	↑ [21]	
Ascorbic acid	↑ [21]							↑ [21]		
2-Amino-3-phosphonoprionic acid	↓ [24]									
Glycerol	↑ [24]									
Malonic Acid	↑ [24]									
Aminalbutanal			↓ [28]							
Guanidinobutanoate			↓ [28]		↓ [28]					
Choline			↓ [29]		↓ [29]					
Ergothioneine						↓ [31]				
N-acetylneuraminate						↓ [31]				
Sedoheptulose-7-phosphate						↓ [31]				
2-Hydroxyheptanoate						↑ [31]				
Glycerate						↑ [31, 34]				
α-Tocopherol						↓ [31, 34]				
Trigonelline						↓ [31, 34]				
Glutarate						↑ [31]				
Dehydroascorbate						↑ [31, 34]				
2-Methylcitrate						↓ [31]				
Erythritol						↑ [31]				
Nicotinamide ribonucleotide						↓ [31]				
Nicotinamide riboside						↓ [31]				
Inositol 1-phosphate						↑ [31]				
2 S,3R-Dihydroxybutyrate						↑ [31]				
3-Formylindole						↑ [31]				
6-Oxopiperidine-2-carboxylate						↑ [34]				
Mannonate						↑ [34]				
Retinol						↓ [34]				
Sphingadienine						↑ [34]				

The table summarizes the changes of different types of metabolites in the HP [21–25], ENT [21], ITG [19, 26–30], MTG [21], MFG [19, 27–30], DLPFC [31–34], SCX [21], MCX [21], CG [21, 22], and CB [19, 21, 25, 28] of AD individuals. Upward (↑) and downward (↓) arrows indicate that the corresponding metabolites exhibited statistically significant increases or decreases in AD versus normal control individuals, or showed significant positive or negative associations with AD diagnosis (*p* < 0.05) in these brain regions, with conclusions derived from the references provided in each cell. Abbreviations: GlcNAc, N-acetylglucosamine; NAA, N-acetylaspartate; GABA, γ-aminobutyric acid; NAG, N-Acetylglutamate; SAH, S-adenosylhomocysteine; SAM, S-adenosylmethionine; NAAG, N-acetyl-aspartyl-glutamate; L-DOPA, Levodopa; LA, linolenic acid; OA, oleic acid; DHA, docosahexaenoic acid; ARA, arachidonic acid; LNA, linoleic acid; EPA, eicosatetraenoic acid; 15-KETE, 15-ketoeicosatetraenoic acid; LysoPC, lysophosphatidylcholine; PC, phosphatidylcholine; aa, diacyl; ae, acyl-ether-containing aliphatic chain; GPC, glycerophosphocholine; GPE, glycerophosphoethanolamine; GPI, glycerophosphoinositol; GPS, glycerophosphoserine; SM, sphingomyelin

As a metabolic neurodegenerative disease, AD manifests hypometabolism of brain glucose, which results in multifactorial pathologies contributing to the disease [35]. Altered abundance of glucose-related metabolites is found in various brain regions, including those with relatively few AD pathology (Table 1) [21]. There was evidence that impaired brain glucose metabolism in AD might be due to the reduction of glycolytic flux and accompanied by decreased levels of neuronal glucose transporter 3 [30]. Meanwhile, alternative fuel sources, including lactate and other sugars, as well as their derivatives, were found to be significantly elevated in AD brains (Table 1) [21, 22]. Since the brain has a high energy demand and it is sensitive to changes in energy fuel supply, neuronal dysfunction and degeneration were also hypothesized to be the subsequent events of deficits in fuel source availability [36]. Thus, some interventions have been recommended to maintain energy metabolism in AD brains, such as ketogenic diets, which produce ketone bodies that replace glucose to fuel the brain [37], and physical exercise, which has been shown to increase glucose utilization [38].

Apart from glucose, lipids also affect brain energy metabolism in a variety of ways [41]. Moreover, the brain is the second most lipidated organ in the body, behind adipose tissue [42]. The presence of lipid changes in the brain has been previously documented in studies examining the pathology of AD [43]. The identification of the ɛ4 allele of the apolipoprotein E (ApoE) gene as the strongest genetic risk factor for AD has further established a link between lipid metabolism and AD [44–46]. Recently, lipidomic and metabolomic studies have consistently demonstrated that diverse classes of lipids are altered or associated with AD neuropathology and cognition in AD brains (Table 1). These include unsaturated fatty acids (FAs) in the MFG, ITG, and CB [19], sphingolipids in the ITG [26], glycerophospholipids in the ITG [26], DLPFC [31, 33], and neocortex [47], and cholesterol-related metabolites in the MFG, ITG [27], and DLPFC [31]. It is worth noting that the levels of certain primary and secondary bile acids (BAs), products of cholesterol metabolism in the liver and gut, which may be transported to the brain, were changed in AD brains [48, 49]. Metabolites that play a pivotal role in lipid metabolism, such as acylcarnitines, which are essential for mitochondrial β-oxidation of FAs [50], also demonstrated a correlation with AD traits [31]. From a mechanistic perspective, lipids are involved in many AD-related processes, including the trafficking and proteolysis of membrane-bound proteins, myelination, neuroinflammation, oxidative stress, and others [42, 45]. Thus, there have been a number of lipid-related AD intervention candidates, including supplementation with long-chain polyunsaturated FAs (PUFAs) [51, 52], statins for cholesterol [53], agonists of lipid-sensitive nuclear receptors [42], and some other treatments rescuing lipid dyshomeostasis [54–57].

In the brain, there is a metabolic connection between neurons and astrocytes via the recycling of neurotransmitters, which is related to the regulation of neurotransmission and brain energy metabolism [58]. It has been suggested that cognitive deficits associated with AD are linked to the brain’s inability to produce acetylcholine because of cholinergic neuron dysfunction [59, 60]. Some metabolites involved in the metabolism of non-cholinergic neurotransmitters have also been linked to AD pathology (Table 1). Reduced hippocampal glutamate (Glu) has been detected in subjects with AD using (1)H magnetic resonance spectroscopy [23]. There were significant differences in γ-aminobutyric acid (GABA) and serotonin (or 5-hydroxytryptamine (5-HT)) metabolism in the ITG between AD individuals and controls, while asymptomatic AD individuals showed lower dopamine (DA) levels in the MFG than controls [28]. A number of AAs, such as arginine and N-acetylaspartate (NAA), which are precursors for the synthesis of different neurotransmitters [61, 62], also showed alterations in diverse regions of AD brains [22, 25, 32]. In addition to cholinesterase inhibitors and Glu receptor agonists that were approved drugs to alleviate AD symptoms, there are other developing therapeutics that regulate neurotransmitters, including GABA and 5-HT [63, 64].

Nowadays, metabolomics changes in the AD brain could be supplemented and verified by transcriptomic and proteomic data, which revealed significant changes in genes and proteins involved in metabolic pathways [65]. Based on regional brain transcriptomic data, alterations in cholesterol metabolism in AD can also be identified by differential expression of genes involved in de novo cholesterol biosynthesis, catabolism, and esterification [27]. In AD brain samples, both alterations in metabolite concentrations and the expression of genes encoding enzymes were observed in the methionine, glutathione, urea, N-acetyl aspartate, and GABA metabolic pathways [29]. The metabolic and enzymatic changes downstream of the Glu/GABA neurotransmitters in the DLPFC of AD individuals were also revealed by integrating proteomics with metabolomics [31]. These gene- and protein- level perturbations of metabolic pathways may also be potential targets for novel therapeutic interventions in AD.

## CSF metabolites associated with AD

In situ characterization of AD-associated neuropathological processes can be accomplished through brain tissue analysis [13]. As an alternative, the CSF is an important biofluid that closely reflects the brain’s extracellular space, with its protein biomarkers enabling the differentiation of AD from other brain disorders [66], yet its utility is hampered by invasive sampling, loss of spatial localization for pathological lesions, and inconsistent component concentrations with local brain tissue due to impaired barrier exchange. Meanwhile, CSF metabolites have also been shown to correlate with AD pathological [67] and predict disease outcomes, making them promising metabolic biomarkers for AD diagnosis [68].

Various CSF metabolites were identified as being individually or commonly associated with biomarkers not only of Aβ and Tau pathologies, but also of neurodegeneration and neuroinflammation (Table 2) [67]. Combination of certain metabolites was found to improve the prediction accuracy of a model incorporating established AD predictors for AD and mild cognitive impairment (MCI) [69]. In subjects with MCI and AD, the cholesterol metabolism in the CSF is significantly disrupted, suggesting cholesterol and its metabolites may serve as sensitive CSF biomarkers of MCI and AD [70]. Besides, the levels of several non-cholesterol sterols and oxysterols in CSF also correlated with AD pathology and could predict cognitive decline (Table 2) [71].

**Table 2:** The association between biofluid metabolites and AD traits

Metabolites				Amyloid			Tangles		Neurodegeneration							Neuroinflammation					Cognition				
CSF	Plasma	Serum	Urine	CSF Aβ42	CSF Aβ 42/40	Aβ PET	CSF *P*-tau		CSF T-tau		FDG PET	HP Vol.	SPARE-AD	NfL	Ng	YKL-40		S100 b	GFAP	sTREM 2	CDR SoB		MM SE	ADAS -Cog13	Mem.
1,2-Dipalmitoyl-GPC (16:0/16:0)				+ [67]			+ [67]		+ [67]					+ [67]	+ [67]	+ [67]		+ [67]	+ [67]	+ [67]					
1-Palmitoyl-2-stearoyl-GPC (16:0/18:0)				+ [67]			+ [67]		+ [67]					+ [67]	+ [67]	+ [67]		+ [67]	+ [67]	+ [67]					
β-Citrylglutamate				+ [67]			+ [67, 69]		+ [67, 69]					+ [67]	+ [67]	+ [67]		+ [67]	+ [67]	+ [67]					
Stearoyl SM (d18:1/18:0)				+ [67]			+ [67]		+ [67]					+ [67]	+ [67]	+ [67]		+ [67]		+ [67]					
1-Palmitoyl-2-oleoyl-GPC (16:0/18:1)				+ [67]			+ [67]		+ [67]					+ [67]	+ [67]	+ [67]		+ [67]		+ [67]					
N-Acetylneuraminate				+ [67]			+ [67, 69]		+ [67, 69]					+ [67]	+ [67]	+ [67]			+ [67]	+ [67]					
N6-Succinyladenosine				+ [67]			+ [67, 69]		+ [67, 69]					+ [67]	+ [67]	+ [67]				+ [67]					
Arabitol/Xylitol				+ [67]			+ [67]		+ [67]					+ [67]	+ [67]	+ [67]				+ [67]					
1-Myristoyl-2-palmitoyl-GPC (14:0/16:0)				+ [67]			+ [67, 69]		+ [67]						+ [67]	+ [67]		+ [67]		+ [67]					
1-Palmitoyl-2-palmitoleoyl-GPC (16:0/16:1)				+ [67]			+ [67]		+ [67, 69]						+ [67]	+ [67]		+ [67]		+ [67]					
Gulonate				+ [67]			+ [67, 69]		+ [67]						+ [67]	+ [67]			+ [67]	+ [67]					
Cysteine				+ [67]			+ [67]		+ [67]					+ [67]	+ [67]	+ [67]									
1-Oleoyl‐GPC (18:1)				+ [67]			+ [69]		+ [69]					+ [67]	+ [67]			+ [67]							
C-Glycosyltryptophan				+ [67]			+ [67, 69]		+ [67, 69]					+ [67]	+ [67]					+ [67]					
Homocarnosine				- [67]			- [67]		- [67]						- [67]	- [67]				- [67]					
Erythronate				+ [67]			+ [67]		+ [67]						+ [67]			+ [67]		+ [67]					
Arabinose				+ [67]			+ [67]		+ [67]						+ [67]				+ [67]	+ [67]					
Creatinine				+ [67]			+ [67]		+ [67]					+ [67]	+ [67]						+ [72]		- [72]		
N-Acetylalanine				+ [67]			+ [67]		+ [67]					+ [67]	+ [67]										
N-Acetylglutamine				+ [67]			+ [67]		+ [67]					+ [67]	+ [67]										
N-Acetyl-isoputreanine				+ [67]			+ [67]		+ [67]					+ [67]	+ [67]										
3-Methylglutarylcarnitine				+ [67]			+ [67]		+ [67]						+ [67]	+ [67]									
1-Stearoyl-2-oleoyl-GPC (18:0/18:1)				+ [67]			+ [67]		+ [67]						+ [67]			+ [67]							
C4-DC				+ [67]			+ [67]		+ [67]						+ [67]					+ [67]					
Arabonate/Xylonate				+ [67]			+ [67]		+ [67]						+ [67]					+ [67]					
2,3-Dihydroxy-2-methylbutyrate				+ [67]			+ [67]		+ [67]						+ [67]					+ [67]					
N-Acetylserine				+ [67]			+ [67]		+ [67]						+ [67]					+ [67]					
N-Acetylthreonine				+ [67]					+ [67, 69]					+ [67]	+ [67]					+ [67]					
Orotate				+ [67]			+ [67]		+ [67]						+ [67]										
Ribonate				+ [67]			+ [67], - [69]		+ [67], - [69]						+ [67]										
3-Methylglutaconate				+ [67]			+ [67]		+ [67]						+ [67]										
N-Acetylarginine				+ [67]			+ [67]		+ [67]						+ [67]										
N-Acetyl-β-alanine				+ [67]			+ [67]		+ [67]						+ [67]										
Carboxyethyl-GABA				+ [67]			+ [67]		+ [67]						+ [67]										
α-Ketoglutaramate				+ [67]			+ [67]		+ [67]						+ [67]										
Cholesterol				+ [67]			+ [69]											+ [67]		+ [67]					
Orotidine				+ [67]										+ [67]	+ [67]					+ [67]					
1-Palmitoyl‐GPC (16:0)									+ [69]					+ [67]	+ [67]			+ [67]							
N-Acetylvaline				+ [67]					+ [67]						+ [67]										
N-Acetylasparagine				+ [67]					+ [67]						+ [67]										
N-Acetylmethionine				+ [67]					+ [67]						+ [67]										
N-Formylmethionine				+ [67]					+ [67]						+ [67]										
GPI				+ [67]										+ [67]						+ [67]					
N-Acetylhistidine							+ [67]		+ [67]						+ [67]										
GlcNAc							+ [67]		+ [67]						+ [67]										
Diglycerol							+ [67]							+ [67]	+ [67]										
SM (d18:2/16:0, d18:1/16:1)														+ [67]				+ [67]		+ [67]					
Palmitoyl SM (d18:1/16:0)														+ [67]				+ [67]		+ [67]					
Dimethylmalonic acid				+ [67]					+ [67]																
Nicotinamide riboside				+ [67]											+ [67]										
NAAG				+ [67]											+ [67]										
Ribitol				+ [67]																+ [67]					
Lysine							+ [67]		+ [67]																
Methylsuccinoylcarnitine							+ [67]								+ [67]										
SM (d18:1/18:1, d18:2/18:0)							+ [67]											+ [67]							
NAG									+ [67]						+ [67]										
1-Palmitoyl-2-docosahexaenoyl-GPC (16:0/22:6)									+ [67]									+ [67]							
1-Arachidonoyl-GPC (20:4n6)														+ [67]	+ [67]										
N-Acetyltaurine				+ [67]																					
5-Methyluridine				+ [67]																					
5,6-Dihydrouridine				+ [67]																					
N6,N6,N6-trimethyllysine				+ [67]																					
4-Acetamidobutanoate				+ [67]																					
β-Hydroxyisovalerate				+ [67]																					
3-(4-Hydroxyphenyl)lactate				+ [67]																					
Allantoin				+ [67]																					
Quinate				+ [67]																					
Leucine							+ [67]																		
Cysteinylglycine							- [67]																		
Gluconate															+ [67]										
Xanthine															+ [67]										
Myo-Inositol																- [67]									
α-Tocopherol																		+ [67]							
SM (d18:2/24:1, d18:1/24:2)																		+ [67]							
1-Stearoyl-GPC (18:0)																		+ [67]							
1-Stearoyl-2-docosahexaenoyl-GPC (18:0/22:6)																		+ [67]							
7-HOCA																				+ [67]					
SM (d18:1/14:0, d16:1/16:0)							+ [69]																		
Desmosterol				+ [71]																	- [71]		+ [71]		
24 S-Hydroxycholesterol							+ [71]		+ [71]																
Sitosterol							+ [71]		+ [71]																
Campesterol									+ [71]																
Adrenaline							+ [73]								+ [73]										
Dopamine							+ [73]																		
Noradrenaline															+ [73]										
Glutamate					- [74]		- [75]					+ [74]											+ [74, 76]		
GDCA											- [77]	- [77]											- [76]	+ [78]	
Sphinganine							- [79]		- [79]																
Oleamide				+ [74]	+ [74]							- [74]											- [74]		
Palmitamide				+ [74]	+ [74]							- [74]											- [74]		
Linoleamide (18:2n6)				+ [74]	+ [74]							- [74]											- [74]		
Stearamide				+ [74]	+ [74]																				
Aspartate				- [74]	- [74]																		+ [74]		
Argininate									- [74]														+ [74]		
γ-Glutamylphenylalanine					- [74]																				
γ-Glutamylglutamate					- [74]																				
SAH					- [74]																				
12,13-DiHOME					- [74]																				
9,10-DiHOME					- [74]																				
PC ae C44:4				+ [4, 75]		+ [80]	+ [75]				- [75]		+ [4]												- [80]
SM C16:0				+ [4], - [26]			+ [26]		+ [26]		- [75]		+ [26]												
C16:1				+ [75]			+ [75]				- [75]		+ [4]											+ [4]	
PC ae C42:4				+ [4]			+ [75]				- [75]		+ [4]												
SM (OH) C14:1				+ [4], - [26]			+ [26]		+ [26]																
C12							+ [75]				- [75]		+ [4]												
C14:1							+ [75]				- [75]													+ [4]	
PC ae C40:3				+ [4, 75]							- [75]														
α-Aminoadipic acid													- [4]											- [4]	
Valine													- [4]											- [4]	
PC ae C36:2				+ [4]																					
SM C20:0																								+ [4]	
C3				+ [26, 75]		- [80]					- [75]		- [26]												+ [80]
SM C18:1							+ [26]		+ [26]				+ [26]												
Serotonin				+ [26]									- [26]												
PC aa C38:4							+ [26]		+ [26]																
PC ae C34:0							+ [26]		+ [26]																
SM (OH) C22:1							+ [26]		+ [26]																
SM (OH) C22:2							+ [26]		+ [26]																
SM C16:1							+ [26]		+ [26]																
PC aa C40:6							- [75]						- [26]												
SM (OH) C24:1							+ [26]																		
SM C26:1							+ [26]																		
Xanthurenic acid																							+ [81]		
TLCA							+ [77]		+ [77]		- [77]	- [77]												+ [78]	
GLCA							+ [77]		+ [77]		- [77]	- [77]												+ [78]	
PC aa C42:0				+ [75]		+ [80]					- [75]														- [80]
PC ae C44:3				+ [75]		+ [80]					- [75]														- [80]
PC ae C44:5				+ [75]		+ [80]					- [75]														- [80]
PC ae C44:6				+ [75]		+ [80]					- [75]														- [80]
Kynurenine				- [75]		- [80]																			+ [80]
LysoPC a C18:2						+ [80]	+ [75]																		- [80]
PC ae C42:3						+ [80]																			- [80]
GCDCA							+ [77]		+ [77]		- [77]	- [77]													
TDCA											- [77]	- [77]													
CA											+ [77]	+ [77]													
TMCA												- [77]													
GUDCA												- [77]													
LysoPC a C16:1				+ [75]			+ [75]				- [75]														
PC ae C42:5				+ [75]			+ [75]				- [75]														
PC aa C36:0				- [75]			- [75]																		
PC aa C42:1				+ [75]							+ [75]														
C5-DC (C6-OH)							+ [75]				- [75]														
C14:2							+ [75]				- [75]														
C18							+ [75]				- [75]														
C18:1							+ [75]				- [75]														
PC aa C34:3							+ [75]				- [75]														
Threonine				+ [75]																					
Asparagine				+ [75]																					
C4				- [75]																					
C8							+ [75]																		
C10							+ [75]																		
C10:2							+ [75]																		
LysoPC a C20:3							+ [75]																		
PC ae C42:2							+ [75]																		
PC ae C38:4							+ [75]																		
PC aa C38:6							- [75]																		
PC ae C40:4							+ [75]																		
PC ae C40:5							+ [75]																		
SM C20:2							+ [75]																		
Arginine											- [75]														
C6 (C4:1-DC)											- [75]														
C16											- [75]														
PC aa C30:0											- [75]														
PC aa C32:0											- [75]														
PC aa C32:1											- [75]														
PC aa C34:4											- [75]														
PC aa C36:5											- [75]														
PC ae C30:0											- [75]														
PC ae C32:1											- [75]														
PC ae C32:2											- [75]														
PC ae C34:1											- [75]														
PC ae C36:0											- [75]														
SM C24:1											- [75]														
Azelaic acid				- [82]					+ [82]			- [82]													
Glutaric acid				+ [82]																					

The table summarizes the association of metabolites from CSF [67, 69, 71, 72], plasma [73–75], serum [4, 26], [76–80] and urine [81, 82] with AD traits. Different colors correspond to various biofluids. “+” and “-” represent positive and negative correlation respectively. The correlation results shown in each cell are derived from the references cited therein. Abbreviations: PET, positron emission tomography; FDG, fluorodeoxyglucose; Vol., volume; SPARE-AD, Spatial Pattern of Abnormalities for Recognition of Early AD; NfL, neurofilament light chain; Ng., neurogranin; YKL-40, chitinase-3-like protein 1; S100b, S100 calcium binding protein b; GFAP, glial fibrillary acidic protein; sTREM2, soluble triggering receptor expressed on myeloid cells 2 CDR-SoB, clinical dementia rating scale sum of boxes; MMSE, mini-mental state examination; ADAS-Cog13, Alzheimer’s Disease Assessment Scale-cognitive subscale 13; Mem., memory; C4-DC, succinylcarnitine; GDCA, chenodeoxycholic acid; 12,13-diHOME, 12,13-dihydroxy-9Z-octadecenoic acid; 9,10-DiHOME, 9,10-dihydroxyoctadec-12-enoic acid; C16:1, hexadecenoylcarnitine; C12, dodecanoylcarnitine; C14:1, tetradecenoylcarnitine; C3, propionylcarnitine; TLCA, taurolithocholic acid; GLCA, glycolithocholic acid; GCDCA, glycochenodeoxycholic acid; TDCA, taurodeoxycholic acid; CA, cholic acid; TMCA, trimethoxycinnamic acid; GUDCA, glycoursodeoxycholic acid; C14:2, tetradecadienoylcarnitine; C18, stearoylcarnitine; C18:1, oleylcarnitine; C4, butyrylcarnitine; C8, octanoylcarnitine; C10, decanoylcarnitine; C10:2, decadienoylcarnitine; C16, palmitoylcarnitine

Among neurotransmitters, several other metabolites are altered in the CSF of AD subjects in addition to those found in AD brains. Noradrenaline and adrenaline were increased, while DA metabolite, homovanillic acid, were reduced in the CSF of AD subjects relative to non-demented controls [72, 83]. Additionally, adjusted multiple regression analyses revealed that DA is positively associated with phosphorylated tau (P-tau), noradrenaline with neurogranin (an index of synaptic damage), and adrenaline with both P-tau and neurogranin, in the CSF (Table 2) [72]. And higher CSF noradrenaline metabolism was found to be sufficient to detect learning declines, implying that the dysregulated noradrenergic-metabolism contribute to AD progression [84].

## Blood metabolite signatures of AD

Diagnostic evaluations of CSF are invasive, especially in elderly people, and require specialized personnel, which makes CSF unsuitable for routine clinical use [85]. Non-invasive blood plasma or serum can be used for the identification of biomarker molecules in clinical practice more effectively and easily than CSF samples [13]. Metabolic profiling in blood could be utilized for accurate diagnosis, such as at early clinical (e.g., MCI) or preclinical stages, as well as monitoring disease progression and therapeutic effectiveness within individuals [86].

### Plasma

In both MCI and AD, non-targeted metabolomics studies detected significantly altered metabolites and metabolic pathways in the plasma. Cognitively normal (CN), MCI, and AD individuals differ in their plasma metabolic pathways, including polyamine metabolism, lysine metabolism, and tryptophan (Trp) metabolism [86]. Based on network modelling, several hubs of metabolic dysregulation were identified in the plasma of AD individuals compared to controls: tyrosine, glycylglycine, glutamine (Gln), and lysophosphatic acid C18:2 [87]. Conjugated BAs, branch-chain AAs, and Glu-related features in the plasma showed significant correlations with clinical stage, cognition, and Aβ deposition [74]. Besides, modules of co-expressed plasma metabolites were associated with amyloid, tau and neurodegeneration (ATN) biomarkers of AD (Table 2) [75]. It is noteworthy that other covariates, such as age, gender, and ApoE genotype, could affect the plasma metabolite network in AD patients as well as the association between plasma metabolite modules and AD endophenotypes [74, 75, 87]. What’s more, the panel of plasma metabolites could predict cognitive decline before clinical symptoms appear in Aβ-positive individuals [88].

There is a high incidence of abnormal lipid metabolism in plasma metabolomics studies of subjects with AD. The metabolism of PC as well as the ratio of PC to lysoPC in plasma distinguish healthy controls (HC) from AD subjects [89, 90]. Altered plasma levels of sphingolipids, including sphingomyelin (SM), have also been observed in AD subjects [91]. Logistic regressions revealed primary fatty amides in plasma associated with amyloid burden in CSF, hippocampal volume, and memory (Table 2) [73]. And there’s more, metabolites in ATN markers-related modules were enriched in lipid pathways [75]. Along with metabolomics, lipidomics were used to comprehensively study lipid in AD [92]. Novel associations between plasma lipids and clinical diagnosis, disease progression, brain atrophy, and disease risk genes, have been identified in nontargeted lipidomics studies of AD (Table 2) [92–94]. A panel of plasma phospholipids discovered using a lipidomic approach showed very high accuracy for predicting either amnestic MCI or AD in the next two or three years [95].

### Serum

Besides metabolites altered in AD brains, such as 5-HT and Trp, many novel biomarkers were identified in the serum of AD subjects, including α-ketoglutarate (α-KG), isocitrate pipecolic acid, pyroglutamate, adenosine, kynurenine (Kyn), and xanthurenic acid [80, 96]. A lipid metabolic disorder was also revealed in the serum samples of AD subjects, involving the metabolism of lysoPC, sphingolipids, and fatty acid amide [85]. Significantly altered serum BAs were found in AD compared to CN and revealed by lower levels of the primary BA, cholic acid (CA), and an increase of secondary BA, deoxycholic acid (DCA) [78]. Additionally, in combination with CSF biomarkers, a number of serum metabolites with early changes were identified in CN participants with pathological CSF Aβ1–42 values compared with CN with no pathological CSF Aβ1–42 values [4].

Metabolic alterations associated with distinct AD endophenotypes may reveal key biological pathways associated with AD. Acylcarnitines and amines are highly associated with AD worse outcomes in blood (Table 2) [97]. Partial correlation analysis indicated that higher serum levels of acylcarnitine, PC, and SM were associated with worse clinical and CSF biomarker measures, while it is the opposite for valine and α-aminoadipic acid (Table 2) [4]. According to spearman’s rank correlation tests, serum glycerophospholipids and sphingolipids are associated with brain atrophy and CSF biomarkers (Aβ42, total tau (T-tau), and P-tau) of AD, and they were connected to signal cascades implicated in AD pathologies (Aβ formation, tau phosphorylation, and apoptosis) (Table 2) [26]. A multivariable regression analysis identified that increased serum propionylcarnitine and Kyn levels were associated with lower cortical Aβ deposition measured by positron emission tomography (PET) and higher memory scores, whereas higher levels of seven PCs were associated with increased Aβ deposition (Table 2) [79]. Besides, serum-based BA metabolites were also found to be associated with “A/T/N” biomarkers of AD, including CSF Aβ1–42, CSF P-tau181, and brain atrophy on T1-weighted magnetic resonance imaging, hypometabolism on fluorodeoxyglucose (FDG) PET, and CSF T-tau (Table 2) [77]. Likewise, the serum metabolome in AD and associations of serum metabolites with AD biomarkers were also influenced by sex and ApoE ε4 status [76].

Serum metabolites could also be screened and combined together to generalize diagnostic and predictive models for AD. The differential serum metabolites in AD were able to distinguish AD from controls and other dementias [98]. In a follow-up study, a molecular signature comprised of 3 serum metabolites predicted progression to AD [99]. In addition, higher serum levels of 3 acylcarnitines could predict reduced incident AD risk after 4.5 years, regardless of age, gender, and education [33]. Nevertheless, the large-scale independent validation of these exploratory biomarker studies in well-characterized samples is critical to determining their clinical utility [12].

## Changes of urinary and salivary metabolome in AD individuals

Since obtaining urine and saliva from participants in clinical studies is relatively convenient and non-invasive, identifying urinary and salivary biomarkers, including metabolites, would be widely applicable. In urine samples, AAs and FAs were significantly different between AD individuals and controls [85]. As well as metabolites associated with glycerolipid and glycerophospholipid metabolism, thermogenesis, and caffeine metabolism, AD urine contained significant differences [100]. In addition, a reduction of metabolites in the Trp pathway, including 5-HT, and an increase in Kyn/Trp ratio indicated a shift to the Kyn metabolic route in urine from AD individuals [80]. Several urine metabolites have also been associated with biomarkers for AD and cognitive performance. Urine dicarboxylic acids, for instance, are linked to CSF tau, CSF Aβ42, and hippocampal volume (Table 2) [82]. The ratio of 3-hydroxypropyl mercapturic acid/creatinine in urine was well correlated with plasma Aβ40/42 and cognitive function (Table 2) [81]. It is also possible to develop a test for diagnosing MCI and AD using urine metabolites [101, 102].

Among the readily available biofluids of human, saliva also exhibits sensitivity to metabolic changes [103, 104]. The concentrations of several metabolites, including those associated with periodontal disease, such as propionate, were found to markedly change in the saliva of AD patients compared to controls [104–106]. Integrating metabolomics and proteomics revealed that the saliva of AD individuals exhibited dysregulation in a variety of metabolites and key enzymes, which involve in tyrosine metabolism, pyruvate metabolism, and glycolysis [107]. The diagnostic model using salivary metabolites could distinguish AD from CN and MCI, making saliva a promising biofluid for discovering biomarkers and studying mechanisms for AD [108]. Salivary metabolite panels, supplemented by other indicators (e.g., cognitive performance and genetic risk factors), robustly predicted the clinical status of AD, reflecting the necessity of combinations of diversiform biomarkers for AD diagnosis [109]. However, multivariate analysis based on metabolites demonstrated a clear separation between AD, other dementias, and HC for serum samples, but not for saliva samples, indicating that serum might be more efficient and suitable for AD diagnosis than saliva [110].

## Gut microbial metabolites involved in AD

It is estimated that trillions of gut microbes exist in the human body, which undergo dynamic changes in response to physiological conditions, encode millions of genes, and produce a wide variety of metabolites [111, 112]. Gut microbial metabolites can derive from bacterial metabolism (short-chain FAs (SCFAs), exopolysaccharides, and AA derivatives), or from bacterial modification of host-derived molecules (secondary BAs) [113–115]. These metabolites can be absorbed through the intestinal epithelium and enter the circulation, travelling to various organs, including the brain [116]. A number of metabolites derived from the microbiota are neuromodulatory, including Trp metabolites, 5-HT, catecholamines, and SCFAs [117]. Importantly, the gut-derived metabolites have been found to influence complex behaviors through impacts on brain functions and affect pathologies associated with aging, such as AD [114, 118, 119].

Metabolites originating from the gut microbiome have been detected in the brain and blood of AD individuals and found to be altered [48, 77, 78]. The relationship between gut microbial metabolites and the development of AD could also be directly investigated by metabolomics analysis of feces [120]. For instance, according to fecal metabolomics profiling, there are significant differences in Trp metabolites, SCFAs, and lithocholic acid levels among AD, amnestic MCI, and HC [121]. The altered gut microbial and fecal metabolites were found to simultaneously distinguish AD and have a relationship with some clinical parameters of AD and inflammatory cytokines [122]. Nowadays, many lines of evidence have indicated that microbiome and its metabolites have an impact on gut and blood-brain barrier integrity, glial and neuronal function, and inflammation in AD pathology [123]. Meanwhile, new directions for research and clinical interventions of AD have been revealed by the role of the gut-brain axis and the regulation of gut microbiota balance [124, 125]. Sodium oligomannate (GV-971), a novel agent approved by China’s National Medical Products Administration for the treatment of mild to moderate AD, has been demonstrated to alter microbial metabolism and reduce neuroinflammation and Aβ deposition [126].

## The role of metabolites in the pathogenesis of AD

The formation of Aβ plaques and NFTs composed of hyperphosphorylated tau in the brain is widely regarded as the two most critical pathological hallmarks of AD, accompanied by chronic neuroinflammation and progressive neuronal degeneration. Dysregulated metabolites and perturbed metabolic pathways have increasingly been recognized as critical contributors to these pathological cascades, offering novel insights into AD pathogenesis.

### Metabolites exert an influence on Aβ and tau pathology

**Fig. 2:**
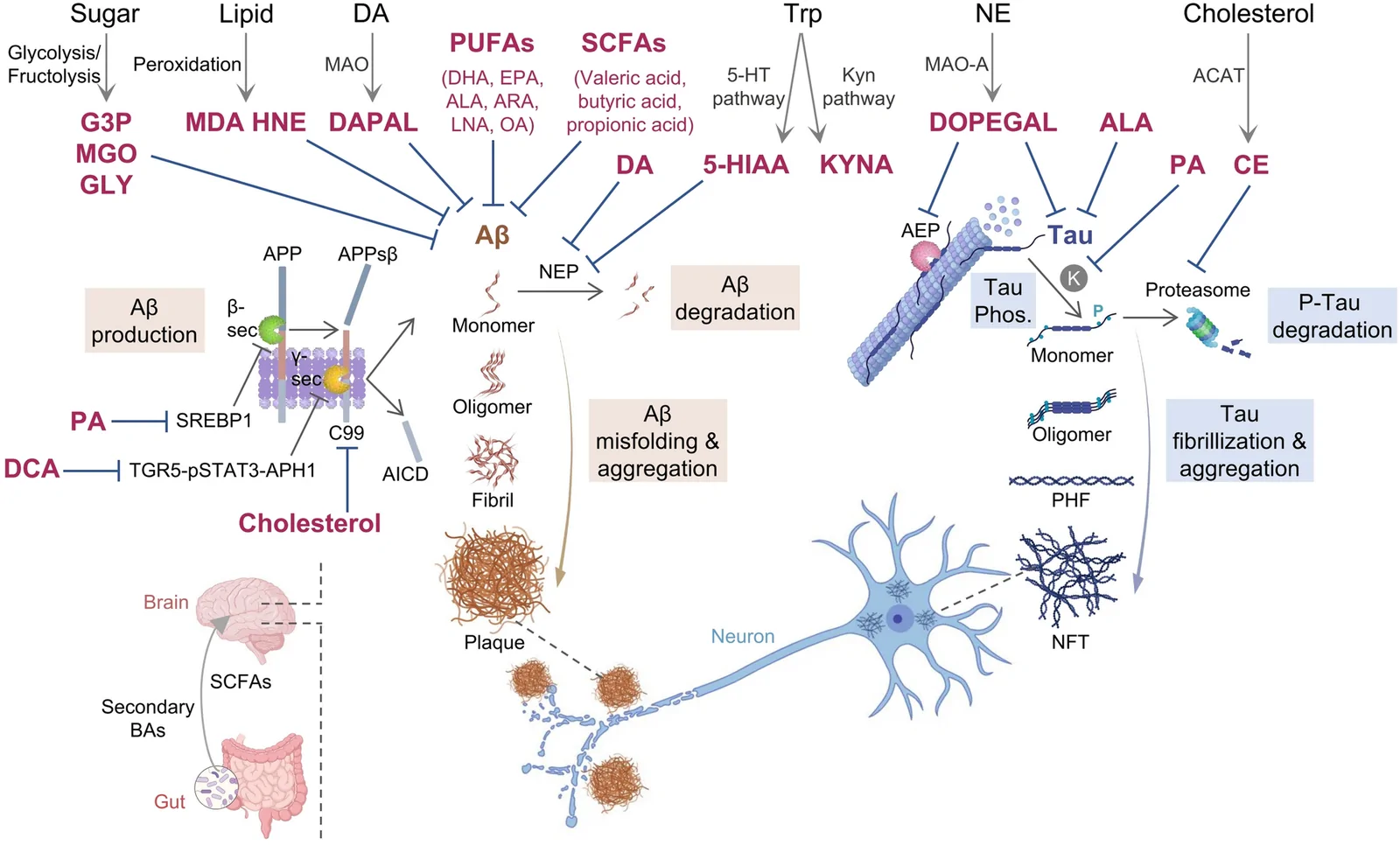
The biochemical links of metabolites and Aβ and tau pathologies. PA-induced SREBP1 activation regulates β-secretase expression [132]. Increased levels of DCA induce the upregulation of TGR5, which promotes Aβ production through a “p-STAT3–APH-1–γ-secretase” pathway [133]. The binding of cholesterol to C99 serves as a cofactor to promote amyloidogenesis [134]. Trp metabolites 5-HIAA and KynA induce NEP expression [141–143]. DA up-regulates NEP expression [144]. Glycation and lipid peroxide metabolites modify Aβ and increase its misfolding [145]. DA metabolite DAPAL stabilizes neurotoxic Aβ oligomers [146]. Select SCFAs (valeric acid, butyric acid, and propionic acid) inhibit the formation of oligomers and fibrils from Aβ peptides [147]. PUFAs DHA, EPA, ALA, ARA, LNA and OA prevent Aβ-fibrillogenesis by direct interactions [148]. DOPEGAL activates AEP, which subsequently cleaves tau, facilitating tau aggregation in the LC of AD [149]. DOPEGAL modifies tau and enhances its fibrillization [14]. PA induces the activation of the main kinases that phosphorylates tau and increases tau accumulation [150]. CE regulate P-tau through the proteasome [151]. ALA inhibits the tau assembly [152]. Figure created with BioRender.com

The concurrent and synergistic accumulation of Aβ plaques and tau aggregates elicits a cascade of deleterious cellular and structural alterations—including synaptic loss, neurodegeneration, and cerebral atrophy—that act as fundamental drivers of the cognitive deficits characteristic of AD [127, 128]. An expanding body of evidence indicates that metabolites are involved in the Aβ and tau pathological processes through a range of biochemical pathways (Fig. 2). First, a number of metabolites play a role in the amyloidogenic pathway, which encompasses the proteolytic processing of amyloid precursor protein (APP) by β-secretase and γ-secretase, resulting in the production of Aβ [129–131]. Palmitic acid (palmitate, PA), a saturated FA that is abundant in the brain, elicits sterol response element binding protein 1 (SREBP1) activation, which can upregulate β-secretase expression and subsequent Aβ genesis [132]. Elevated levels of DCA facilitate the expression of Takeda G protein-coupled receptor 5 (TGR5), which activates signal transducer and activator of transcription 3 (STAT3) to promote anterior pharynx defective-1 (Aph1) transcription, leading to increased γ-secretase activity to produce Aβ [133]. A binding site for cholesterol has been identified within the transmembrane C-terminal domain (C99) of APP, and there is evidence to suggest that this binding may play a role in the amyloidogenic pathway and lipid abnormalities in AD [134, 135]. Besides, cholesterol provides optimal conditions for β- and γ-secretase activity by forming lipid rafts, which promotes the production of Aβ [136, 137]. The depletion of cholesterol may enhance the nonamyloidogenic pathway, thereby reducing Aβ generation [138–140].

Secondly, certain metabolites are associated with the clearance of Aβ, which is crucial for maintaining Aβ homeostasis [130, 153]. Trp metabolites 5-hydroxyindole-acetic acid (5-HIAA) and kynurenic acid (KynA) have been found to induce brain or neuronal expression of neprilysin (NEP) [141–143], which is one of the principal Aβ-degrading proteases [154, 155]. DA has been identified as a NEP up-regulator in the anterior cortex, thereby modulating Aβ dynamics in vivo [144].

Thirdly, metabolites can affect the misfolding and aggregation of Aβ into a range of states, including monomers, oligomers, fibrils, and plaques [130]. Glycation metabolites (glyceraldehyde-3-phosphate (G3P), methylglyoxal (MGO), and glyceraldehyde (GLY)) and lipid peroxidation products (malondialdehyde (MDA) and hydroxynonenal (HNE))increase Aβ misfolding by modifying its specific amino acid residues, as identified through MS analyses [145, 156, 157]. The monoamine oxidase (MAO) product of DA, 3,4-dihydroxyphenylacetaldehyde (DOPAL), a toxic and highly reactive aldehyde, has been shown to promote the formation of stable Aβ oligomers by inducing more spherical aggregate morphologies [146]. In contrast, particular SCFAs (valeric acid, butyric acid, and propionic acid) were observed to inhibit Aβ misfolding into neurotoxic oligomers [147]. PUFAs docosahexaenoic acid (DHA), eicosatetraenoic acid (EPA), α-linolenic acid (ALA), arachidonic acid (ARA), linoleic acid (LNA), and oleic acid (OA) are capable of directly interacting with Aβ40 and Aβ42 peptides and prevent Aβ-fibrillogenesis [148].

Similarly, some metabolites have been identified as affecting tau pathology in AD (Fig. 2). The locus coeruleus (LC), the primary source of the neurotransmitter norepinephrine (NE) in the central nervous system, is specifically vulnerable to early tau deposition in AD [158, 159]. 3,4-Dihydroxyphenylglycolaldehyde (DOPEGAL), a metabolite of NE produced by MAO-A, activates asparagine endopeptidase (AEP), which cleaves tau and abolishes its microtubule assembly function. This results in tau aggregation and LC neurodegeneration [149, 160]. Moreover, DOPEGAL can interact with tau independently of AEP, reacting directly with the primary amine on the Lys353 residue of tau to modify the protein and thereby stimulating its aggregation and propagation [14]. PA was found to induce AD-related tau phosphorylation by activating signaling cascades that impact the kinase activity and increase tau acetylation, leading to tau accumulation [150]. Cholesteryl esters (CE), derived from cholesterol by acyl-CoA cholesterol acyltransferase (ACAT), affect P-tau levels by regulating its degradation through the proteasome [151]. Besides, ALA was observed to inhibit the aggregation of tau and maintain its monomeric form [152]. This evidence together shows that metabolites influence the core pathologies of AD, suggesting the potential of harnessing these processes to boost resistance and resilience against AD pathology.

### Metabolites act as mediators and regulators in the neuroinflammation-neurodegeneration axis

**Fig. 3:**
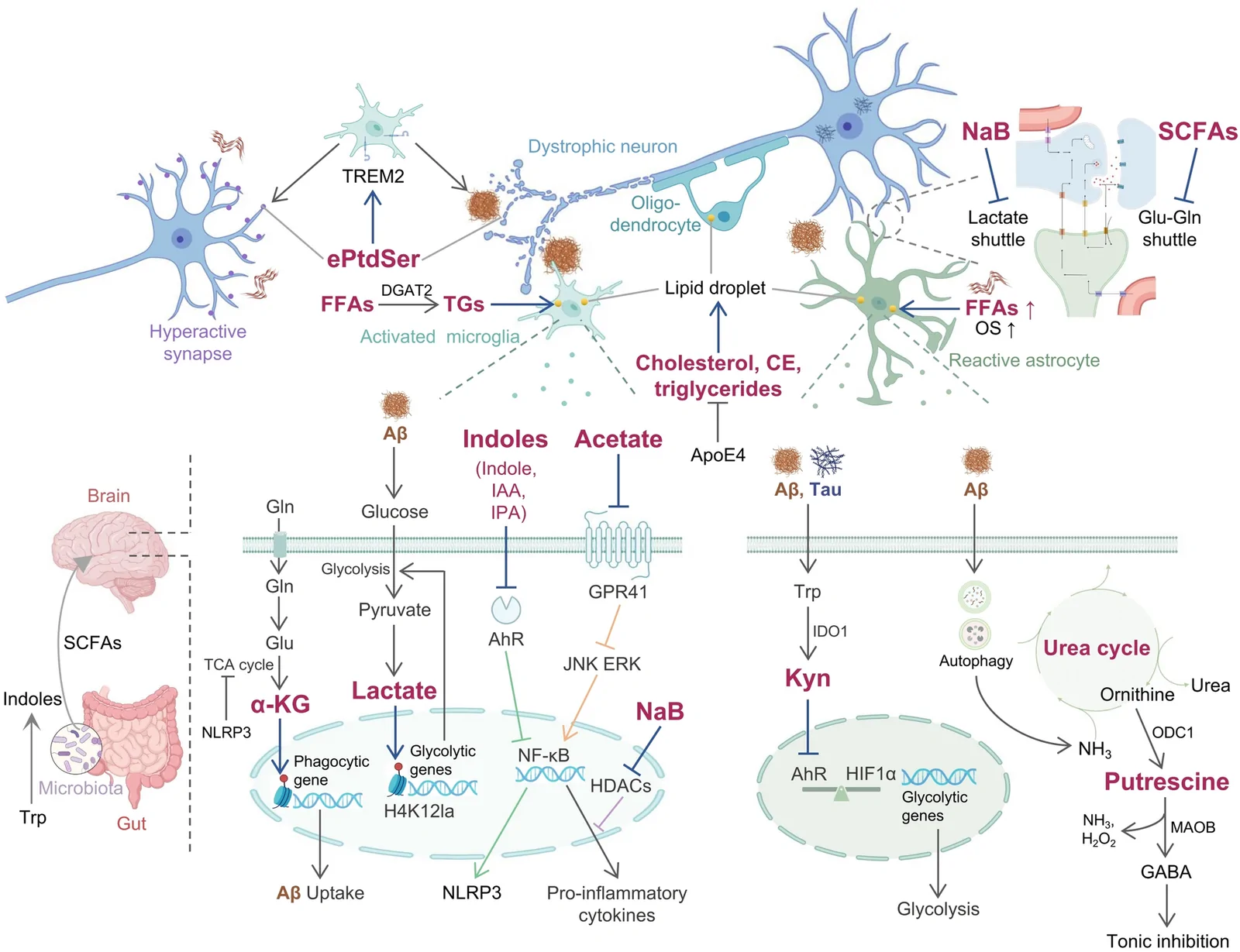
Metabolites serve as mediators and regulators of glial functions and neuroinflammation in AD. Microglia engulf ePtdSer⁺ hyperactive synapses and migrate toward Aβ plaques surrounded by ePtdSer derived from dystrophic neurons, with both processes mediated by TREM2 [163, 164]. In microglia: loss of NLRP3 can increase Gln and Glu metabolism and α-KG generation, which enhances phagocytic gene transcription and Aβ clearance [165]; there is a positive feedback loop comprising active glycolysis, lactate production, H4K12la modification, and glycolytic gene expression [166]; indoles (indole, indole-3-acetic acid (IAA) and indole-3-propionic acid (IPA)) upregulate the production of AhR, inhibit the activation of the NF-κB signal pathway and the formation of the NLRP3 inflammasome [167]; acetate exerted anti-neuroinflammatory effects via the upregulation of GPR41 and suppression of the ERK/JNK/NF-κB pathway [168]; NaB demonstrated the capacity to reduce the expression of inflammatory factors as an inhibitor of HDAC [169]. SCFAs facilitate the Glu-Gln shuttle, and NaB promotes the lactate shuttle between astrocytes and neurons [170, 171]. In astrocytes, Increased IDO1-mediated Kyn production disrupts the balance between AhR and hypoxia-inducible factor 1-α (HIF1α), leading to a reduction in glycolysis [172]; Aβ induces upregulated urea cycle, producing aberrant putrescine by ornithine decarboxylase-1 (ODC1) and its byproducts NH_3_, H_2_O_2_, and GABA [17]. Aberrant formation and accumulation of LDs in glial cells are induced by multiple stressors under AD pathological conditions [173–175]. ApoE4 is linked to lipid metabolism dysregulation in microglia, astrocytes, and oligodendrocytes [176–178]. Figure created with BioRender.com

There is a mutually reinforcing relationship between pathogenic protein depositions and neuroinflammation, which exacerbates neurodegeneration in AD [161]. Immune cells in the brain, specifically microglia and astrocytes, serve as key mediators of neuroinflammation [162]. It has been established that metabolites play a part in the normal functioning of glial cells and are related to neuroinflammation (Fig. 3).

Microglia assume both beneficial and detrimental roles in AD [179]. These roles are contingent upon a multitude of factors [180]. Moreover, certain metabolites are implicated in mediating or regulating these processes (Fig. 3). In the early stages of AD, the externalized phosphatidylserine (ePtdSer) underlies the detection and engulfment of hyperactive synapses by microglia via triggering receptor expressed on myeloid cells 2 (TREM2) [164]. Similarly, ePtdSer also functions as an initiator of TREM2-dependent microglial phagocytosis of Aβ plaques [163]. Increased Gln utilization and α-KG levels, with α-KG modulating the epigenetic landscape to drive phagocytic gene transcription, can consequently enhance microglial Aβ phagocytosis [165]. This process is induced by the inhibition of NOD-like receptor family pyrin domain-containing 3 (NLRP3) [165], which was also shown to be regulated by Gln metabolism in microglia in AD [181].

As AD progresses, the continuous deposition of Aβ leads to the chronic activation of the microglia as well as the impairment of microglial phagocytic function, which have been indicated as disease-promoting factors [179, 182]. A positive feedback loop involving lactate production by active glycolysis, histone H4 lysine 12 lactylation (H4K12la), and glycolytic gene expression in microglia exacerbates its activation in AD [166, 183]. Indoles, the main microbial catabolites of Trp [184], can activate the aryl hydrocarbon receptor (AhR) signal pathway, inhibit the formation of NLRP3 inflammasomes, and then reduce microglia-mediated neuroinflammation in AD mice [167]. SCFAs have been shown to regulate neuroinflammation by acting as ligands for G protein-coupled receptors (GPRs) [111, 185, 186]. For instance, acetate might attenuate microglia-mediated neuroinflammation via the upregulation of GPR41 and the subsequent inhibition of the c-Jun N-terminal kinase (ERK)/extracellular signal-regulated kinase (JNK)/ nuclear factor-κB (NF-κB) pathway in AD [168]. Besides, SCFAs also act as histone deacetylase (HDAC) inhibitors, which exhibit immunosuppressive and neuroprotective effects [187]. The interactions between sodium butyrate (NaB), as one of SCFAs, and HDACs, results in the activation of anti-inflammatory response genes and the inhibition of pro-inflammatory genes [169]. Microglial lipid droplet (LD) accumulation is also closely associated with neuroinflammation and linked to the upregulation of Diacylglycerol O-acyltransferase 2 (DGAT2), an enzyme that mediates the conversion of free FAs (FFAs) to triacylglycerols (TGs) [173, 175].

Astrocytes, as key regulators of neurotransmission and brain homeostasis, can become reactive in AD and contribute to neurodegeneration [188]. The roles of astrocytes in AD are also associated with metabolic function and could be modulated by metabolites (Fig. 3). The increased conversion of Trp to Kyn that ensues upon the activation of astrocytic indoleamine-2,3-dioxygenase 1 (IDO1) by Aβ and tau oligomers curtails astrocytic glycolysis and the metabolic support of neurons [172]. The upregulation of the astrocytic urea cycle, which is induced by Aβ degradation, aberrantly produces putrescine and its byproducts NH_3_, H_2_O_2_, and GABA, leading to oxidative stress and neurodegeneration in AD [17]. SCFAs has been demonstrated to enhance the metabolic coupling between astrocytes and neurons, including the Glu-Gln shuttle, which may be impaired in AD [189], and alleviate AD pathology [171]. Among them, NaB was revealed to improve the mitochondrial function of astrocytes and the astrocyte-neuron lactate shuttle, which may contribute to the alleviation of cognitive impairment in AD mice [170]. Notably, astrocytic LD formation and aberrant accumulation occur under AD pathological conditions [175]. Aβ oligomers directly drive astrocytic LD formation, while multiple metabolic and pathological stressors, including excess FFAs and oxidative stress, induce aberrant LD accumulation [174, 175].

### The effects of AD risk genes on metabolome

Given its pronounced genetic underpinnings, AD presents an early-onset, dominantly inherited subtype, while the late-onset subtype is also influenced by genetic elements [190, 191]. Genetic risk factors for AD encompass fully penetrant mutations in APP, presenilin 1 and 2 (PSEN1/2) (linked to early-onset familial AD (FAD)), rare variants in susceptibility genes like TREM2, sortilin-related receptor (SORL1), and ATP binding cassette subfamily A member 7 (ABCA7), and common high-risk variants of ApoE (a major determinant of late-onset AD) [1, 192, 193]. Intriguingly, most of these genes are functionally related to metabolism, especially lipid metabolism [46, 190, 194–197]. The genetic variants of AD manifest unique metabolic disruptions [34, 76, 198], providing compelling evidence that AD risk genes impact the metabolome.

PSEN plays a role in neuronal lipid homeostasis, encompassing the modulation of the levels of cholesterol and SM [194], and PSEN1 mutations were associated with changes in astrocyte metabolism, such as Glu uptake [198]. TREM2 exerts a significant influence on cerebral glucose metabolism [199], and functions as a pivotal regulator of lipid metabolism [195]. In AD individuals with TREM2 risk variants and TREM2-deficient AD-like mice, microglia show abnormal autophagy linked to impaired mammalian target of rapamycin (mTOR) activation and metabolism [200]. TREM2-deficient microglia can engulf myelin debris, yet their inability to eliminate myelin cholesterol causes the accumulation of CE [201]. AD-relevant SORL1 loss of function leads to a reduction in ApoE and clusterin (CLU) specifically in neuron and altered lipid profiles [202]. When SORL1 is depleted, it reprograms energy metabolism from glucose to FAs, with FA oxidation boosting ATP supply yet failing to reduce ROS [203]. Deficiency of ABCA7 may also contribute to the risk of AD by altering mitochondria-associated phospholipids [204].

The ApoE gene is the strongest AD genetic risk factor, with the ε4 allele raising risk 2-4-fold over the ε3 allele [205]. There is mounting evidence that ApoE4-dependent abnormalities in glial lipid metabolism are associated with AD (Fig. 3). A correlation has been identified between the ApoE4/4 genotype and the presence of abundant LD-containing microglia in the brains of AD individuals. These microglia may secrete factors that induce tau pathology and neuronal apoptosis [178]. ApoE4 can facilitate the abnormal accumulation of unsaturated triacylglycerides and LDs in human iPSC-derived astrocytes [176]. It also upregulates astrocyte cholesterol synthesis and matrisome associated with chemotaxis, neuroinflammation, and lipid metabolism in the brain [206]. Moreover, there is an anomalous accumulation of cholesterol in oligodendrocytes, accompanied by a decrease in myelination in the brains of individuals with the ApoE4 allele [177]. The influence of ApoE genotype on different glial cells and lipid metabolism in these cells may have both similarities and certain specificity. It is necessary to further study the consequences resulting from these influences and their interconnections in AD. In addition, a complex regulatory feedback loop may exist between TREM2 and ApoE, considering that ApoE serves as a ligand for TREM2 [207, 208], which might have a key role in lipid homeostasis and the states of AD-associated glia.

## Studying the metabolome in AD: clinical translation and mechanistic elucidation

**Fig. 4:**
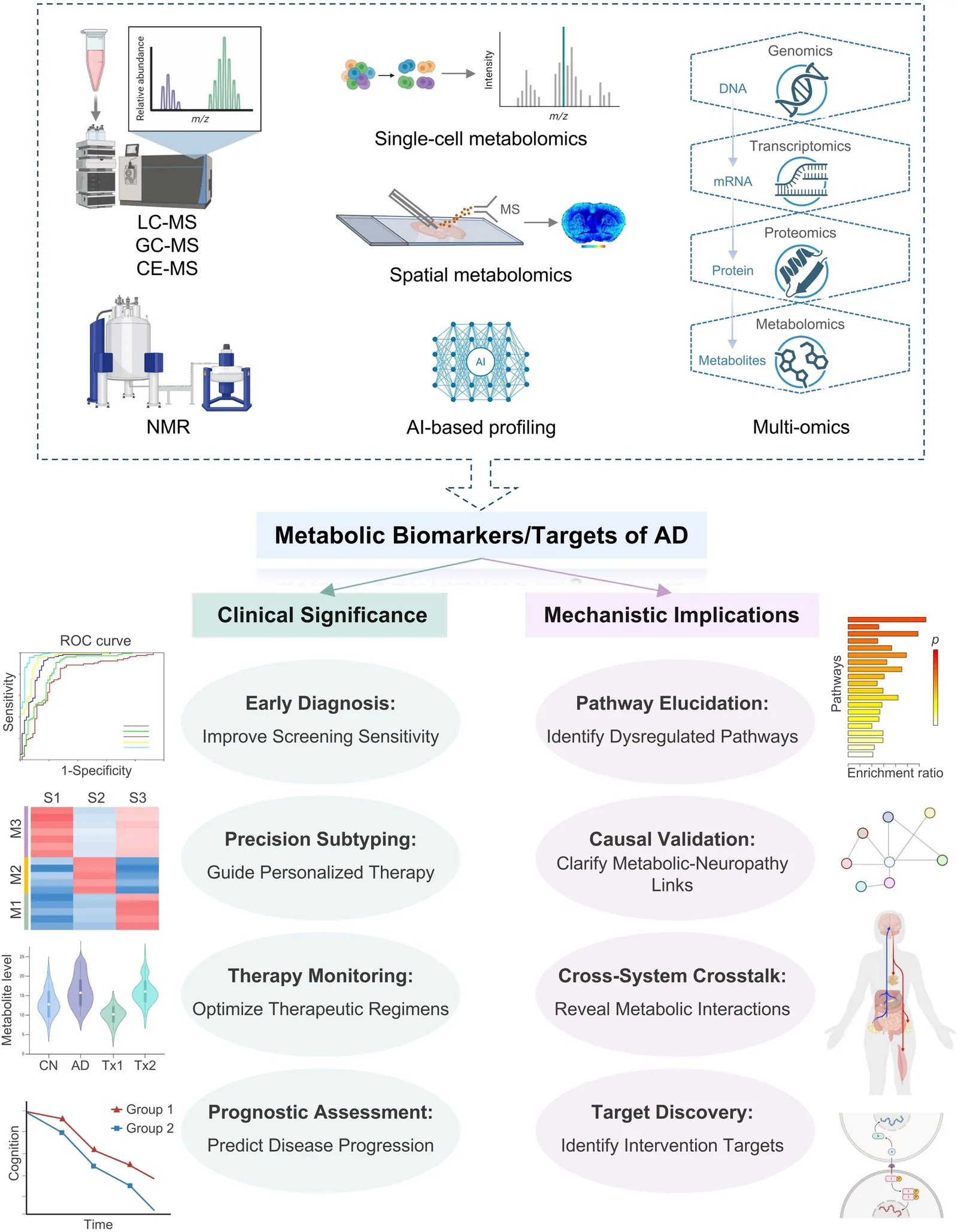
Studying metabolomics in AD: clinical and mechanistic significances. Contemporary metabolomics research uses four core platforms (LC-MS, GC-MS, NMR spectroscopy, CE-MS), with recent advancements including single-cell, spatial, and AI-enhanced metabolic profiling, and emerging integrative approaches with other omics. In AD, metabolomics identifies metabolic biomarkers and targets to support clinical translation and mechanistic studies: clinically, it may facilitate early diagnosis, precision subtyping, treatment efficacy monitoring, and prognosis prediction; mechanistically, it helps elucidate dysregulated metabolic pathways, validate metabolism-neuropathology links, reveal cross-system crosstalk, and identify potential intervention targets. Abbreviations: m/z, mass to charge ratio; DNA, deoxyribonucleic acid; mRNA, messenger ribonucleic acid; ROC, receiver operating characteristic; S1/2/3, subtype 1/2/3; M1/2/3, module 1/2/3; Tx1/2, treatment 1/2; *p*, p value. Figure created with BioRender.com

Contemporary metabolomics leverages four core analytical platforms: liquid chromatography-MS (LC-MS) for broad metabolite coverage, gas chromatography-MS (GC-MS) for volatile compounds, nuclear magnetic resonance (NMR) spectroscopy for structural elucidation and quantification, and capillary electrophoresis-MS (CE-MS) for charged species [209]. It also encompasses two complementary analytical strategies—untargeted and targeted metabolomics—where the former enables unbiased, comprehensive metabolite profiling with semi-quantitative capacity for discovering novel metabolic changes, and the latter focuses on predefined metabolites to achieve accurate quantification with higher sensitivity and reproducibility. The field has advanced through single-cell techniques [210, 211], spatial mapping [212], and artificial intelligence (AI)-driven profiling [213]. Emerging directions integrate flux analysis at single-cell resolution [214] and combine metabolomics with other omics for comprehensive biological interpretation [75]. Principal contributions of metabolomics in AD include identifying metabolic biomarkers and key metabolic targets, facilitating both clinical translation and mechanistic understanding (Fig. 4).

In terms of clinical value, metabolomics can support early diagnosis of AD by uncovering preclinical metabolic signals and distinguish AD from other dementia via disease-specific metabolic profiles, thereby improving the sensitivity and specificity of disease screening [69, 88, 95, 98]. It may also facilitate precision subtyping by stratifying disease subtypes based on differences in metabolic profiles, providing a scientific basis for guiding the personalized treatment strategies tailored to individual characteristics. In AD drug development, metabolomics could enable real-time monitoring of treatment efficacy via dynamic metabolite tracking [215], supporting rigorous efficacy assessment in clinical trials and timely optimization of therapeutic regimens. Furthermore, it may be utilized to improve prognostic evaluation in AD by predicting disease progression and recovery potential via metabolic signatures, thus providing a supplementary objective reference for clinical decision-making.

In the realm of basic mechanism value, studying metabolome plays a crucial role in pathway elucidation, enabling the precise identification of core dysregulated metabolic pathways that underpin AD pathogenesis [80, 97]. It enables the identification of potential links between metabolites, core neuropathological hallmarks, and clinical phenotypes of AD, thereby enriching the metabolic perspective on AD progression [67, 73, 79]. Moreover, the metabolome reveals systemic metabolic crosstalk mechanisms in AD, such as the gut-brain axis, where gut microbial metabolites modulate brain function through endocrine and neural signaling pathways [216, 217]. It also facilitates the discovery of potential intervention targets in AD, including metabolites and their associated genes, enzymes, and signaling molecules, which lay the groundwork for the development of novel therapies [172, 218–220].

Collectively, these metabolome-focused investigations are anticipated to deepen our understanding of AD pathogenesis, facilitate the refinement of clinical diagnostic and therapeutic strategies, and advance targeted interventions.

## Limitations

Although abundant metabolomic findings have deepened our understanding of AD-related metabolic disturbances, several crucial limitations remain to be addressed. First, most brain metabolomic data are acquired from post-mortem tissues of late-stage AD patients, which fail to capture dynamic metabolic alterations during preclinical and MCI stages and hinder the identification of early-AD metabolic signatures. Although biofluid samples can partially reflect early metabolic changes across disease stages, they cannot fully recapitulate the authentic metabolic characteristics of brain tissue. Second, current AD metabolomic studies are predominantly cross-sectional and correlation-based, with systematic translational validation in human cohorts remaining insufficient to delineate the causal relationship between metabolic dysregulation and AD pathogenesis. Third, untargeted metabolomics, the mainstream exploratory approach in current AD metabolic research, lacks unified community-standard experimental and analytical pipelines, rendering it prone to inherent technical variability and statistical noise that compromises the reproducibility of metabolic profiles [221]. In addition, uncontrolled confounding factors including age, sex and ApoE genotype further exacerbate inter-study heterogeneity, weakening the robustness of identified metabolic signatures. Fourth, despite the emerging progress of cell-resolved multi-omics techniques, most clinical AD metabolic investigations still depend on bulk-level metabolomic detection, and a systematic metabolic regulatory network underlying AD pathogenesis has not been fully constructed.

## Conclusion and perspectives

Increasing metabolomics‑based studies have focused on AD. Metabolic anomalies within the central nervous system and peripheral tissues strongly support the viewpoint that AD can be regarded as a metabolic‑related disease [222]. Multiple metabolites linked to disease progression, pathological features and cognitive decline have been identified across diverse biological samples and regarded as promising AD biomarkers. Moreover, metabolomics data can facilitate the classification of AD metabolic subtypes, therapeutic response monitoring and prognosis evaluation, providing metabolic references for personalized diagnosis and treatment of AD. To address the above‑mentioned limitations, longitudinal and dynamic metabolic profiling covering preclinical and prodromal AD stages is urgently needed to identify early metabolic signatures and clarify the temporal causal relationship between metabolic dysregulation and AD pathological progression. Notably, targeted metabolomic validation is required to verify candidate metabolites screened by untargeted approaches and consolidate their credibility. Rigorous control of confounding factors and large‑cohort validation will further improve the reliability and cross-study comparability of metabolic profiles, reducing technical and population heterogeneity. Benefiting from the advancement of high-resolution analytical approaches, emerging spatial and single-cell metabolomics techniques complement conventional bulk metabolomics methods, enabling precise dissection of region-specific metabolic alterations and cellular-level metabolic heterogeneity in AD. Further mechanistic exploration and multi‑omics integration with transcriptomics and proteomics will help unravel the regulatory networks of AD‑related metabolites, laying a solid foundation for future individualized diagnosis and precise treatment of AD.

## Supplementary Information

Below is the link to the electronic supplementary material.

Supplementary Material 1

## Data Availability

No datasets were generated or analysed during the current study.
