# Supplementary material for: Deciphering the metabolome in Alzheimer’s disease
Source: Mol Neurodegener. 2026 Jul 31;21:71. doi: 10.1186/s13024-026-00982-w (PMC13729493; doi:10.1186/s13024-026-00982-w)
Supplement: Supplementary file 1 [file 13024_2026_982_MOESM1_ESM.pdf]

HP	total	expected	hits	Raw p	Holm p	FDR
Galactose Metabolism	38	1.29	6	0.00122	0.12	0.12
Arginine and Proline Metabolism	52	1.76	6	0.00639	0.62	0.313
Urea Cycle	28	0.95	4	0.0127	1	0.415
Spermidine and Spermine Biosynthesis	18	0.611	3	0.0206	1	0.435
Aspartate Metabolism	35	1.19	4	0.0274	1	0.435
Homocysteine Degradation	9	0.305	2	0.0347	1	0.435
Warburg Effect	57	1.93	5	0.039	1	0.435
Malate-Aspartate Shuttle	10	0.339	2	0.0424	1	0.435
Glycine and Serine Metabolism	59	2	5	0.0444	1	0.435
Inositol Phosphate Metabolism	24	0.814	3	0.0444	1	0.435
Phosphatidylethanolamine Biosynthesis	12	0.407	2	0.0596	1	0.454
Phenylalanine and Tyrosine Metabolism	27	0.916	3	0.0599	1	0.454
Glucose-Alanine Cycle	13	0.441	2	0.069	1	0.454
Phospholipid Biosynthesis	29	0.984	3	0.0714	1	0.454
Glutamate Metabolism	48	1.63	4	0.0746	1	0.454
Inositol Metabolism	30	1.02	3	0.0775	1	0.454
Phosphatidylcholine Biosynthesis	14	0.475	2	0.0788	1	0.454
Ammonia Recycling	31	1.05	3	0.0839	1	0.457
Gluconeogenesis	33	1.12	3	0.0973	1	0.502
Beta-Alanine Metabolism	34	1.15	3	0.104	1	0.511
Valine, Leucine and Isoleucine Degradation	59	2	4	0.134	1	0.613
Catecholamine Biosynthesis	20	0.679	2	0.145	1	0.613
Glutathione Metabolism	20	0.679	2	0.145	1	0.613
Sphingolipid Metabolism	40	1.36	3	0.15	1	0.613
Methionine Metabolism	42	1.43	3	0.167	1	0.615
Carnitine Synthesis	22	0.747	2	0.169	1	0.615
Transfer of Acetyl Groups into Mitochondria	22	0.747	2	0.169	1	0.615
Glycolysis	23	0.78	2	0.182	1	0.636
Glycerolipid Metabolism	25	0.848	2	0.207	1	0.682
Tyrosine Metabolism	70	2.38	4	0.209	1	0.682
Cysteine Metabolism	26	0.882	2	0.219	1	0.694
Biotin Metabolism	8	0.271	1	0.242	1	0.74
Lactose Degradation	9	0.305	1	0.268	1	0.74
De Novo Triacylglycerol Biosynthesis	9	0.305	1	0.268	1	0.74
Lysine Degradation	30	1.02	2	0.271	1	0.74
Starch and Sucrose Metabolism	31	1.05	2	0.284	1	0.74
Fructose and Mannose Degradation	31	1.05	2	0.284	1	0.74
Amino Sugar Metabolism	33	1.12	2	0.309	1	0.74
D-Arginine and D-Ornithine Metabolism	11	0.373	1	0.317	1	0.74
Glycerol Phosphate Shuttle	11	0.373	1	0.317	1	0.74
Trehalose Degradation	11	0.373	1	0.317	1	0.74
Cardiolipin Biosynthesis	11	0.373	1	0.317	1	0.74
Taurine and Hypotaurine Metabolism	12	0.407	1	0.341	1	0.776
Thyroid hormone synthesis	13	0.441	1	0.363	1	0.809
Propanoate Metabolism	42	1.43	2	0.422	1	0.92
Alanine Metabolism	17	0.577	1	0.447	1	0.931
Phosphatidylinositol Phosphate Metabolism	17	0.577	1	0.447	1	0.931
Mitochondrial Electron Transport Chain	19	0.645	1	0.484	1	0.968
Lactose Synthesis	19	0.645	1	0.484	1	0.968
Nucleotide Sugars Metabolism	20	0.679	1	0.502	1	0.984
Pantothenate and CoA Biosynthesis	21	0.713	1	0.519	1	0.998
Oxidation of Branched Chain Fatty Acids	26	0.882	1	0.597	1	1
Phytanic Acid Peroxisomal Oxidation	26	0.882	1	0.597	1	1
Tryptophan Metabolism	59	2	2	0.607	1	1
Selenoamino Acid Metabolism	27	0.916	1	0.611	1	1
Pentose Phosphate Pathway	29	0.984	1	0.638	1	1
Folate Metabolism	29	0.984	1	0.638	1	1
Citric Acid Cycle	32	1.09	1	0.675	1	1
Nicotinate and Nicotinamide Metabolism	35	1.19	1	0.708	1	1
Fatty Acid Biosynthesis	35	1.19	1	0.708	1	1
Purine Metabolism	73	2.48	2	0.725	1	1
Histidine Metabolism	42	1.43	1	0.773	1	1
Pyruvate Metabolism	47	1.59	1	0.81	1	1
Pyrimidine Metabolism	57	1.93	1	0.868	1	1
Arachidonic Acid Metabolism	67	2.27	1	0.909	1	1

ENT	total	expected	hits	Raw p	Holm p	FDR
Galactose Metabolism	38	0.834	6	9.51E-05	0.00932	0.00932
Arginine and Proline Metabolism	52	1.14	6	0.000575	0.0558	0.0282
Urea Cycle	28	0.615	4	0.00253	0.243	0.0826
Ammonia Recycling	31	0.681	4	0.00372	0.353	0.0911
Transfer of Acetyl Groups into Mitochondria	22	0.483	3	0.0108	1	0.212
Inositol Phosphate Metabolism	24	0.527	3	0.0138	1	0.225
Glutamate Metabolism	48	1.05	4	0.0179	1	0.228
Malate-Aspartate Shuttle	10	0.22	2	0.0186	1	0.228
Inositol Metabolism	30	0.659	3	0.0254	1	0.261
Phosphatidylethanolamine Biosynthesis	12	0.263	2	0.0266	1	0.261
Warburg Effect	57	1.25	4	0.0319	1	0.267
Gluconeogenesis	33	0.725	3	0.0327	1	0.267
Glycine and Serine Metabolism	59	1.3	4	0.0357	1	0.269
Alanine Metabolism	17	0.373	2	0.0513	1	0.359
Methionine Metabolism	42	0.922	3	0.0605	1	0.396
Purine Metabolism	73	1.6	4	0.0696	1	0.426
Glycolysis	23	0.505	2	0.0882	1	0.508
Glycerolipid Metabolism	25	0.549	2	0.102	1	0.554
Selenoamino Acid Metabolism	27	0.593	2	0.116	1	0.575
Phospholipid Biosynthesis	29	0.637	2	0.131	1	0.575
Pentose Phosphate Pathway	29	0.637	2	0.131	1	0.575
Starch and Sucrose Metabolism	31	0.681	2	0.146	1	0.575
Fructose and Mannose Degradation	31	0.681	2	0.146	1	0.575
Citric Acid Cycle	32	0.703	2	0.154	1	0.575
Beta-Alanine Metabolism	34	0.747	2	0.169	1	0.575
Aspartate Metabolism	35	0.768	2	0.177	1	0.575
Thiamine Metabolism	9	0.198	1	0.182	1	0.575
Phenylacetate Metabolism	9	0.198	1	0.182	1	0.575
Homocysteine Degradation	9	0.198	1	0.182	1	0.575
Lactose Degradation	9	0.198	1	0.182	1	0.575
De Novo Triacylglycerol Biosynthesis	9	0.198	1	0.182	1	0.575
D-Arginine and D-Ornithine Metabolism	11	0.242	1	0.218	1	0.593
Glycerol Phosphate Shuttle	11	0.242	1	0.218	1	0.593
Trehalose Degradation	11	0.242	1	0.218	1	0.593
Cardiolipin Biosynthesis	11	0.242	1	0.218	1	0.593
Sphingolipid Metabolism	40	0.878	2	0.218	1	0.593
Glucose-Alanine Cycle	13	0.285	1	0.252	1	0.667
Phosphatidylcholine Biosynthesis	14	0.307	1	0.269	1	0.693
Pyruvate Metabolism	47	1.03	2	0.276	1	0.694
Phosphatidylinositol Phosphate Metabolism	17	0.373	1	0.316	1	0.738
Spermidine and Spermine Biosynthesis	18	0.395	1	0.332	1	0.738
Butyrate Metabolism	19	0.417	1	0.347	1	0.738
Mitochondrial Electron Transport Chain	19	0.417	1	0.347	1	0.738
Lactose Synthesis	19	0.417	1	0.347	1	0.738
Ethanol Degradation	19	0.417	1	0.347	1	0.738
Nucleotide Sugars Metabolism	20	0.439	1	0.361	1	0.738
Glutathione Metabolism	20	0.439	1	0.361	1	0.738
Riboflavin Metabolism	20	0.439	1	0.361	1	0.738
Pantothenate and CoA Biosynthesis	21	0.461	1	0.376	1	0.751
Carnitine Synthesis	22	0.483	1	0.39	1	0.764
Cysteine Metabolism	26	0.571	1	0.443	1	0.826
Phenylalanine and Tyrosine Metabolism	27	0.593	1	0.455	1	0.826
Mitochondrial Beta-Oxidation of Short Chain Saturated Fatty Acids	27	0.593	1	0.455	1	0.826
Mitochondrial Beta-Oxidation of Medium Chain Saturated Fatty Acids	27	0.593	1	0.455	1	0.826
Mitochondrial Beta-Oxidation of Long Chain Saturated Fatty Acids	28	0.615	1	0.468	1	0.833
Amino Sugar Metabolism	33	0.725	1	0.525	1	0.919
Nicotinate and Nicotinamide Metabolism	35	0.768	1	0.546	1	0.94
Porphyrin Metabolism	40	0.878	1	0.596	1	1
Propanoate Metabolism	42	0.922	1	0.614	1	1
Histidine Metabolism	42	0.922	1	0.614	1	1
Fatty acid Metabolism	43	0.944	1	0.623	1	1
Pyrimidine Metabolism	57	1.25	1	0.728	1	1
Tryptophan Metabolism	59	1.3	1	0.741	1	1
Bile Acid Biosynthesis	65	1.43	1	0.775	1	1
Tyrosine Metabolism	70	1.54	1	0.8	1	1

ITG	total	expected	hits	Raw p	Holm p	FDR
Glutamate Metabolism	48	1.01	6	0.000277	0.0271	0.0182
Arginine and Proline Metabolism	52	1.09	6	0.000435	0.0422	0.0182
Glutathione Metabolism	20	0.419	4	0.000557	0.0535	0.0182
Glycine and Serine Metabolism	59	1.24	6	0.000877	0.0834	0.0215
Methionine Metabolism	42	0.88	5	0.00126	0.118	0.0246
Urea Cycle	28	0.587	4	0.00211	0.196	0.0345
Alpha Linolenic Acid and Linoleic Acid Metabolism	17	0.356	3	0.00447	0.411	0.0577
Aspartate Metabolism	35	0.734	4	0.0049	0.446	0.0577
Spermidine and Spermine Biosynthesis	18	0.377	3	0.00529	0.477	0.0577
Malate-Aspartate Shuttle	10	0.21	2	0.017	1	0.167
Ammonia Recycling	31	0.65	3	0.0245	1	0.218
Glucose-Alanine Cycle	13	0.272	2	0.0284	1	0.229
Tryptophan Metabolism	59	1.24	4	0.0305	1	0.229
Phosphatidylcholine Biosynthesis	14	0.293	2	0.0327	1	0.229
Alanine Metabolism	17	0.356	2	0.0471	1	0.308
Purine Metabolism	73	1.53	4	0.0602	1	0.369
Betaine Metabolism	21	0.44	2	0.0692	1	0.399
Carnitine Synthesis	22	0.461	2	0.0751	1	0.409
Methylhistidine Metabolism	4	0.0838	1	0.0814	1	0.42
Cysteine Metabolism	26	0.545	2	0.101	1	0.493
Beta-Alanine Metabolism	34	0.713	2	0.157	1	0.664
Arachidonic Acid Metabolism	67	1.4	3	0.161	1	0.664
Nicotinate and Nicotinamide Metabolism	35	0.734	2	0.165	1	0.664
Homocysteine Degradation	9	0.189	1	0.174	1	0.664
Lactose Degradation	9	0.189	1	0.174	1	0.664
Tyrosine Metabolism	70	1.47	3	0.176	1	0.664
Pyruvaldehyde Degradation	10	0.21	1	0.192	1	0.696
Trehalose Degradation	11	0.231	1	0.209	1	0.713
Histidine Metabolism	42	0.88	2	0.219	1	0.713
Taurine and Hypotaurine Metabolism	12	0.251	1	0.226	1	0.713
Phosphatidylethanolamine Biosynthesis	12	0.251	1	0.226	1	0.713
Lactose Synthesis	19	0.398	1	0.334	1	0.974
Warburg Effect	57	1.19	2	0.338	1	0.974
Catecholamine Biosynthesis	20	0.419	1	0.348	1	0.974
Ubiquinone Biosynthesis	20	0.419	1	0.348	1	0.974
Pantothenate and CoA Biosynthesis	21	0.44	1	0.362	1	0.985
Transfer of Acetyl Groups into Mitochondria	22	0.461	1	0.376	1	0.985
Glycolysis	23	0.482	1	0.389	1	0.985
Bile Acid Biosynthesis	65	1.36	2	0.4	1	0.985
Estrone Metabolism	24	0.503	1	0.402	1	0.985
Phenylalanine and Tyrosine Metabolism	27	0.566	1	0.44	1	1
Phospholipid Biosynthesis	29	0.608	1	0.464	1	1
Folate Metabolism	29	0.608	1	0.464	1	1
Lysine Degradation	30	0.629	1	0.475	1	1
Amino Sugar Metabolism	33	0.692	1	0.509	1	1
Androgen and Estrogen Metabolism	33	0.692	1	0.509	1	1
Gluconeogenesis	33	0.692	1	0.509	1	1
Galactose Metabolism	38	0.796	1	0.56	1	1
Porphyrin Metabolism	40	0.838	1	0.579	1	1
Sphingolipid Metabolism	40	0.838	1	0.579	1	1
Propanoate Metabolism	42	0.88	1	0.597	1	1
Pyruvate Metabolism	47	0.985	1	0.639	1	1
Valine, Leucine and Isoleucine Degradation	59	1.24	1	0.724	1	1

MTG	total	expected	hits	Raw p	Holm p	FDR
Gluconeogenesis	33	0.659	4	0.00326	0.32	0.27
Galactose Metabolism	38	0.758	4	0.00551	0.535	0.27
Glycolysis	23	0.459	3	0.00934	0.897	0.305
Fructose and Mannose Degradation	31	0.619	3	0.0214	1	0.414
Warburg Effect	57	1.14	4	0.023	1	0.414
Amino Sugar Metabolism	33	0.659	3	0.0253	1	0.414
Glutamate Metabolism	48	0.958	3	0.0663	1	0.844
Transfer of Acetyl Groups into Mitochondria	22	0.439	2	0.0689	1	0.844
Inositol Phosphate Metabolism	24	0.479	2	0.0804	1	0.875
Glycine and Serine Metabolism	59	1.18	3	0.108	1	0.94
Pentose Phosphate Pathway	29	0.579	2	0.111	1	0.94
Inositol Metabolism	30	0.599	2	0.118	1	0.94
Starch and Sucrose Metabolism	31	0.619	2	0.125	1	0.94
Lactose Degradation	9	0.18	1	0.167	1	1
Purine Metabolism	73	1.46	3	0.174	1	1
Malate-Aspartate Shuttle	10	0.2	1	0.183	1	1
D-Arginine and D-Ornithine Metabolism	11	0.22	1	0.2	1	1
Trehalose Degradation	11	0.22	1	0.2	1	1
Phosphatidylethanolamine Biosynthesis	12	0.24	1	0.216	1	1
Glucose-Alanine Cycle	13	0.259	1	0.232	1	1
Phosphatidylcholine Biosynthesis	14	0.279	1	0.247	1	1
Arginine and Proline Metabolism	52	1.04	2	0.278	1	1
Alanine Metabolism	17	0.339	1	0.292	1	1
Lactose Synthesis	19	0.379	1	0.321	1	1
Nucleotide Sugars Metabolism	20	0.399	1	0.334	1	1
Glutathione Metabolism	20	0.399	1	0.334	1	1
Threonine and 2-Oxobutanoate Degradation	20	0.399	1	0.334	1	1
Carnitine Synthesis	22	0.439	1	0.361	1	1
Glycerolipid Metabolism	25	0.499	1	0.4	1	1
Phenylalanine and Tyrosine Metabolism	27	0.539	1	0.424	1	1
Urea Cycle	28	0.559	1	0.436	1	1
Phospholipid Biosynthesis	29	0.579	1	0.447	1	1
Ammonia Recycling	31	0.619	1	0.47	1	1
Citric Acid Cycle	32	0.639	1	0.481	1	1
Beta-Alanine Metabolism	34	0.679	1	0.502	1	1
Aspartate Metabolism	35	0.699	1	0.512	1	1
Porphyrin Metabolism	40	0.798	1	0.561	1	1
Sphingolipid Metabolism	40	0.798	1	0.561	1	1
Methionine Metabolism	42	0.838	1	0.579	1	1
Pyruvate Metabolism	47	0.938	1	0.621	1	1
Pyrimidine Metabolism	57	1.14	1	0.694	1	1
Tryptophan Metabolism	59	1.18	1	0.706	1	1
Bile Acid Biosynthesis	65	1.3	1	0.742	1	1

MFG	total	expected	hits	Raw p	Holm p	FDR
Catecholamine Biosynthesis	20	0.319	5	7.07E-06	0.000693	0.000693
Phosphatidylcholine Biosynthesis	14	0.224	3	0.00109	0.106	0.046
Methylhistidine Metabolism	4	0.0639	2	0.00141	0.135	0.046
Alpha Linolenic Acid and Linoleic Acid Metabolism	17	0.271	3	0.00199	0.189	0.0486
Methionine Metabolism	42	0.671	4	0.00337	0.317	0.0523
Tyrosine Metabolism	70	1.12	5	0.00344	0.32	0.0523
Betaine Metabolism	21	0.335	3	0.00373	0.343	0.0523
Glutamate Metabolism	48	0.766	3	0.0372	1	0.377
Glutathione Metabolism	20	0.319	2	0.0384	1	0.377
Ubiquinone Biosynthesis	20	0.319	2	0.0384	1	0.377
Carnitine Synthesis	22	0.351	2	0.0459	1	0.409
Estrone Metabolism	24	0.383	2	0.0538	1	0.439
Glycine and Serine Metabolism	59	0.942	3	0.0628	1	0.473
Nicotinate and Nicotinamide Metabolism	35	0.559	2	0.105	1	0.733
Homocysteine Degradation	9	0.144	1	0.135	1	0.809
Lactose Degradation	9	0.144	1	0.135	1	0.809
Histidine Metabolism	42	0.671	2	0.142	1	0.809
Pyruvaldehyde Degradation	10	0.16	1	0.149	1	0.809
Trehalose Degradation	11	0.176	1	0.163	1	0.809
Taurine and Hypotaurine Metabolism	12	0.192	1	0.177	1	0.809
Phosphatidylethanolamine Biosynthesis	12	0.192	1	0.177	1	0.809
Glucose-Alanine Cycle	13	0.208	1	0.19	1	0.809
Thyroid hormone synthesis	13	0.208	1	0.19	1	0.809
Arginine and Proline Metabolism	52	0.83	2	0.2	1	0.816
Tryptophan Metabolism	59	0.942	2	0.242	1	0.948
Spermidine and Spermine Biosynthesis	18	0.287	1	0.253	1	0.955
Lactose Synthesis	19	0.303	1	0.266	1	0.964
Pantothenate and CoA Biosynthesis	21	0.335	1	0.289	1	0.982
Arachidonic Acid Metabolism	67	1.07	2	0.291	1	0.982
Transfer of Acetyl Groups into Mitochondria	22	0.351	1	0.301	1	0.983
Glycolysis	23	0.367	1	0.312	1	0.987
Cysteine Metabolism	26	0.415	1	0.345	1	1
Phenylalanine and Tyrosine Metabolism	27	0.431	1	0.356	1	1
Phospholipid Biosynthesis	29	0.463	1	0.377	1	1
Androgen and Estrogen Metabolism	33	0.527	1	0.417	1	1
Gluconeogenesis	33	0.527	1	0.417	1	1
Galactose Metabolism	38	0.607	1	0.464	1	1
Sphingolipid Metabolism	40	0.639	1	0.482	1	1
Pyruvate Metabolism	47	0.75	1	0.539	1	1
Steroid Biosynthesis	48	0.766	1	0.547	1	1
Warburg Effect	57	0.91	1	0.611	1	1
Purine Metabolism	73	1.17	1	0.705	1	1

DLPFC	total	expected	hits	Raw p	Holm p	FDR
Glycine and Serine Metabolism	59	2.18	9	0.000157	0.0153	0.0153
Urea Cycle	28	1.03	5	0.00276	0.267	0.135
Aspartate Metabolism	35	1.29	5	0.00753	0.723	0.246
Lysine Degradation	30	1.11	4	0.0217	1	0.422
Ammonia Recycling	31	1.14	4	0.0242	1	0.422
Spermidine and Spermine Biosynthesis	18	0.665	3	0.0258	1	0.422
Nicotinate and Nicotinamide Metabolism	35	1.29	4	0.0363	1	0.508
Methionine Metabolism	42	1.55	4	0.0645	1	0.785
Phenylalanine and Tyrosine Metabolism	27	0.997	3	0.0737	1	0.785
Glucose-Alanine Cycle	13	0.48	2	0.0801	1	0.785
Arginine and Proline Metabolism	52	1.92	4	0.12	1	1
Alanine Metabolism	17	0.628	2	0.127	1	1
Methylhistidine Metabolism	4	0.148	1	0.14	1	1
Galactose Metabolism	38	1.4	3	0.161	1	1
Catecholamine Biosynthesis	20	0.739	2	0.166	1	1
Glutathione Metabolism	20	0.739	2	0.166	1	1
Betaine Metabolism	21	0.775	2	0.18	1	1
Carnitine Synthesis	22	0.812	2	0.193	1	1
Inositol Phosphate Metabolism	24	0.886	2	0.221	1	1
Glutamate Metabolism	48	1.77	3	0.259	1	1
Biotin Metabolism	8	0.295	1	0.261	1	1
Phenylacetate Metabolism	9	0.332	1	0.288	1	1
Inositol Metabolism	30	1.11	2	0.305	1	1
Malate-Aspartate Shuttle	10	0.369	1	0.315	1	1
D-Arginine and D-Ornithine Metabolism	11	0.406	1	0.34	1	1
Amino Sugar Metabolism	33	1.22	2	0.347	1	1
Beta-Alanine Metabolism	34	1.26	2	0.36	1	1
Valine, Leucine and Isoleucine Degradation	59	2.18	3	0.374	1	1
Tryptophan Metabolism	59	2.18	3	0.374	1	1
Thyroid hormone synthesis	13	0.48	1	0.389	1	1
Propanoate Metabolism	42	1.55	2	0.466	1	1
Histidine Metabolism	42	1.55	2	0.466	1	1
Phosphatidylinositol Phosphate Metabolism	17	0.628	1	0.475	1	1
Tyrosine Metabolism	70	2.58	3	0.486	1	1
Lactose Synthesis	19	0.702	1	0.514	1	1
Purine Metabolism	73	2.7	3	0.515	1	1
Nucleotide Sugars Metabolism	20	0.739	1	0.532	1	1
Threonine and 2-Oxobutanoate Degradation	20	0.739	1	0.532	1	1
Glycerolipid Metabolism	25	0.923	1	0.614	1	1
Cysteine Metabolism	26	0.96	1	0.629	1	1
Oxidation of Branched Chain Fatty Acids	26	0.96	1	0.629	1	1
Pyrimidine Metabolism	57	2.1	2	0.635	1	1
Warburg Effect	57	2.1	2	0.635	1	1
Selenoamino Acid Metabolism	27	0.997	1	0.643	1	1
Phospholipid Biosynthesis	29	1.07	1	0.669	1	1
Folate Metabolism	29	1.07	1	0.669	1	1
Starch and Sucrose Metabolism	31	1.14	1	0.694	1	1
Fructose and Mannose Degradation	31	1.14	1	0.694	1	1
Retinol Metabolism	35	1.29	1	0.738	1	1
Sphingolipid Metabolism	40	1.48	1	0.785	1	1
Arachidonic Acid Metabolism	67	2.47	1	0.926	1	1

SCX	total	expected	hits	Raw p	Holm p	FDR
Galactose Metabolism	38	0.417	4	0.000481	0.0471	0.0471
Glycolysis	23	0.252	2	0.0245	1	0.785
Phospholipid Biosynthesis	29	0.318	2	0.0379	1	0.785
Starch and Sucrose Metabolism	31	0.34	2	0.0428	1	0.785
Fructose and Mannose Degradation	31	0.34	2	0.0428	1	0.785
Gluconeogenesis	33	0.362	2	0.0481	1	0.785
Lactose Degradation	9	0.0988	1	0.0949	1	0.823
De Novo Triacylglycerol Biosynthesis	9	0.0988	1	0.0949	1	0.823
D-Arginine and D-Ornithine Metabolism	11	0.121	1	0.115	1	0.823
Glycerol Phosphate Shuttle	11	0.121	1	0.115	1	0.823
Trehalose Degradation	11	0.121	1	0.115	1	0.823
Cardiolipin Biosynthesis	11	0.121	1	0.115	1	0.823
Phosphatidylethanolamine Biosynthesis	12	0.132	1	0.125	1	0.823
Warburg Effect	57	0.626	2	0.126	1	0.823
Ketone Body Metabolism	13	0.143	1	0.134	1	0.823
Glucose-Alanine Cycle	13	0.143	1	0.134	1	0.823
Phosphatidylcholine Biosynthesis	14	0.154	1	0.144	1	0.83
Mitochondrial Electron Transport Chain	19	0.209	1	0.191	1	0.979
Lactose Synthesis	19	0.209	1	0.191	1	0.979
Nucleotide Sugars Metabolism	20	0.22	1	0.2	1	0.979
Transfer of Acetyl Groups into Mitochondria	22	0.242	1	0.218	1	1
Inositol Phosphate Metabolism	24	0.263	1	0.235	1	1
Glycerolipid Metabolism	25	0.274	1	0.244	1	1
Urea Cycle	28	0.307	1	0.269	1	1
Pentose Phosphate Pathway	29	0.318	1	0.277	1	1
Inositol Metabolism	30	0.329	1	0.285	1	1
Amino Sugar Metabolism	33	0.362	1	0.309	1	1
Beta-Alanine Metabolism	34	0.373	1	0.317	1	1
Fatty Acid Biosynthesis	35	0.384	1	0.325	1	1
Sphingolipid Metabolism	40	0.439	1	0.363	1	1
Glutamate Metabolism	48	0.527	1	0.419	1	1
Arginine and Proline Metabolism	52	0.571	1	0.445	1	1
Pyrimidine Metabolism	57	0.626	1	0.477	1	1
Purine Metabolism	73	0.801	1	0.567	1	1

MCX	total	expected	hits	Raw p	Holm p	FDR
Galactose Metabolism	38	0.455	5	3.92E-05	0.00384	0.00384
Glycolysis	23	0.275	3	0.00203	0.197	0.0557
Inositol Phosphate Metabolism	24	0.287	3	0.00231	0.222	0.0557
Inositol Metabolism	30	0.359	3	0.00445	0.422	0.0557
Lactose Degradation	9	0.108	2	0.00452	0.425	0.0557
De Novo Triacylglycerol Biosynthesis	9	0.108	2	0.00452	0.425	0.0557
Starch and Sucrose Metabolism	31	0.371	3	0.00489	0.45	0.0557
Fructose and Mannose Degradation	31	0.371	3	0.00489	0.45	0.0557
Gluconeogenesis	33	0.395	3	0.00585	0.527	0.0557
Glycerol Phosphate Shuttle	11	0.132	2	0.00682	0.607	0.0557
Trehalose Degradation	11	0.132	2	0.00682	0.607	0.0557
Cardiolipin Biosynthesis	11	0.132	2	0.00682	0.607	0.0557
Mitochondrial Electron Transport Chain	19	0.228	2	0.0201	1	0.141
Lactose Synthesis	19	0.228	2	0.0201	1	0.141
Transfer of Acetyl Groups into Mitochondria	22	0.263	2	0.0266	1	0.163
Warburg Effect	57	0.683	3	0.0266	1	0.163
Glycerolipid Metabolism	25	0.299	2	0.0339	1	0.195
Urea Cycle	28	0.335	2	0.0418	1	0.219
Phospholipid Biosynthesis	29	0.347	2	0.0446	1	0.219
Pentose Phosphate Pathway	29	0.347	2	0.0446	1	0.219
Amino Sugar Metabolism	33	0.395	2	0.0565	1	0.264
Sphingolipid Metabolism	40	0.479	2	0.0797	1	0.355
Thiamine Metabolism	9	0.108	1	0.103	1	0.44
D-Arginine and D-Ornithine Metabolism	11	0.132	1	0.125	1	0.49
Arginine and Proline Metabolism	52	0.623	2	0.125	1	0.49
Phosphatidylethanolamine Biosynthesis	12	0.144	1	0.135	1	0.51
Pyrimidine Metabolism	57	0.683	2	0.146	1	0.51
Glucose-Alanine Cycle	13	0.156	1	0.146	1	0.51
Phosphatidylcholine Biosynthesis	14	0.168	1	0.156	1	0.528
Alanine Metabolism	17	0.204	1	0.187	1	0.572
Phosphatidylinositol Phosphate Metabolism	17	0.204	1	0.187	1	0.572
Spermidine and Spermine Biosynthesis	18	0.216	1	0.196	1	0.572
Vitamin B6 Metabolism	19	0.228	1	0.206	1	0.572
Nucleotide Sugars Metabolism	20	0.24	1	0.216	1	0.572
Catecholamine Biosynthesis	20	0.24	1	0.216	1	0.572
Glutathione Metabolism	20	0.24	1	0.216	1	0.572
Riboflavin Metabolism	20	0.24	1	0.216	1	0.572
Betaine Metabolism	21	0.251	1	0.226	1	0.576
Sulfate/Sulfite Metabolism	22	0.263	1	0.235	1	0.576
Carnitine Synthesis	22	0.263	1	0.235	1	0.576
Cysteine Metabolism	26	0.311	1	0.272	1	0.605
Oxidation of Branched Chain Fatty Acids	26	0.311	1	0.272	1	0.605
Phytanic Acid Peroxisomal Oxidation	26	0.311	1	0.272	1	0.605
Plasmalogen Synthesis	26	0.311	1	0.272	1	0.605
Selenoamino Acid Metabolism	27	0.323	1	0.281	1	0.612
Folate Metabolism	29	0.347	1	0.298	1	0.636
Ammonia Recycling	31	0.371	1	0.316	1	0.658
Citric Acid Cycle	32	0.383	1	0.324	1	0.662
Beta-Alanine Metabolism	34	0.407	1	0.341	1	0.67
Nicotinate and Nicotinamide Metabolism	35	0.419	1	0.349	1	0.67
Aspartate Metabolism	35	0.419	1	0.349	1	0.67
Propanoate Metabolism	42	0.503	1	0.404	1	0.732
Methionine Metabolism	42	0.503	1	0.404	1	0.732
Histidine Metabolism	42	0.503	1	0.404	1	0.732
Pyruvate Metabolism	47	0.563	1	0.44	1	0.769
Steroid Biosynthesis	48	0.575	1	0.447	1	0.769
Glutamate Metabolism	48	0.575	1	0.447	1	0.769
Glycine and Serine Metabolism	59	0.707	1	0.519	1	0.848
Valine, Leucine and Isoleucine Degradation	59	0.707	1	0.519	1	0.848
Tryptophan Metabolism	59	0.707	1	0.519	1	0.848
Tyrosine Metabolism	70	0.838	1	0.583	1	0.936
Purine Metabolism	73	0.874	1	0.599	1	0.946

CG	total	expected	hits	Raw p	Holm p	FDR
Amino Sugar Metabolism	33	0.626	4	0.00267	0.262	0.148
Warburg Effect	57	1.08	5	0.00314	0.305	0.148
Galactose Metabolism	38	0.721	4	0.00453	0.435	0.148
Glycolysis	23	0.436	3	0.00806	0.766	0.198
Phenylalanine and Tyrosine Metabolism	27	0.512	3	0.0127	1	0.229
Urea Cycle	28	0.531	3	0.014	1	0.229
Fructose and Mannose Degradation	31	0.588	3	0.0186	1	0.253
Gluconeogenesis	33	0.626	3	0.022	1	0.253
Glucose-Alanine Cycle	13	0.247	2	0.0235	1	0.253
Aspartate Metabolism	35	0.664	3	0.0258	1	0.253
Mitochondrial Electron Transport Chain	19	0.36	2	0.0481	1	0.428
Glutathione Metabolism	20	0.379	2	0.0528	1	0.431
Glutamate Metabolism	48	0.91	3	0.0583	1	0.439
Arginine and Proline Metabolism	52	0.986	3	0.0709	1	0.479
Inositol Phosphate Metabolism	24	0.455	2	0.0734	1	0.479
Glycerolipid Metabolism	25	0.474	2	0.0789	1	0.483
Cysteine Metabolism	26	0.493	2	0.0845	1	0.487
Glycine and Serine Metabolism	59	1.12	3	0.0959	1	0.5
Phospholipid Biosynthesis	29	0.55	2	0.102	1	0.5
Pentose Phosphate Pathway	29	0.55	2	0.102	1	0.5
Inositol Metabolism	30	0.569	2	0.108	1	0.505
Starch and Sucrose Metabolism	31	0.588	2	0.114	1	0.509
Beta-Alanine Metabolism	34	0.645	2	0.133	1	0.568
Homocysteine Degradation	9	0.171	1	0.159	1	0.599
Lactose Degradation	9	0.171	1	0.159	1	0.599
De Novo Triacylglycerol Biosynthesis	9	0.171	1	0.159	1	0.599
Malate-Aspartate Shuttle	10	0.19	1	0.175	1	0.603
D-Arginine and D-Ornithine Metabolism	11	0.209	1	0.191	1	0.603
Glycerol Phosphate Shuttle	11	0.209	1	0.191	1	0.603
Trehalose Degradation	11	0.209	1	0.191	1	0.603
Cardiolipin Biosynthesis	11	0.209	1	0.191	1	0.603
Taurine and Hypotaurine Metabolism	12	0.228	1	0.206	1	0.613
Phosphatidylethanolamine Biosynthesis	12	0.228	1	0.206	1	0.613
Ketone Body Metabolism	13	0.247	1	0.222	1	0.638
Phosphatidylcholine Biosynthesis	14	0.265	1	0.236	1	0.662
Alanine Metabolism	17	0.322	1	0.28	1	0.761
Lactose Synthesis	19	0.36	1	0.307	1	0.798
Tryptophan Metabolism	59	1.12	2	0.309	1	0.798
Nucleotide Sugars Metabolism	20	0.379	1	0.321	1	0.806
Pantothenate and CoA Biosynthesis	21	0.398	1	0.334	1	0.818
Transfer of Acetyl Groups into Mitochondria	22	0.417	1	0.347	1	0.829
Tyrosine Metabolism	70	1.33	2	0.388	1	0.905
Purine Metabolism	73	1.38	2	0.409	1	0.932
Folate Metabolism	29	0.55	1	0.431	1	0.959
Lysine Degradation	30	0.569	1	0.442	1	0.962
Ammonia Recycling	31	0.588	1	0.453	1	0.964
Citric Acid Cycle	32	0.607	1	0.463	1	0.966
Nicotinate and Nicotinamide Metabolism	35	0.664	1	0.494	1	0.989
Fatty Acid Biosynthesis	35	0.664	1	0.494	1	0.989
Sphingolipid Metabolism	40	0.758	1	0.542	1	1
Propanoate Metabolism	42	0.796	1	0.56	1	1
Methionine Metabolism	42	0.796	1	0.56	1	1
Histidine Metabolism	42	0.796	1	0.56	1	1
Pyrimidine Metabolism	57	1.08	1	0.675	1	1
Valine, Leucine and Isoleucine Degradation	59	1.12	1	0.688	1	1
Arachidonic Acid Metabolism	67	1.27	1	0.735	1	1

CB	total	expected	hits	Raw p	Holm p	FDR
Urea Cycle	28	0.531	4	0.00142	0.139	0.139
Galactose Metabolism	38	0.721	4	0.00453	0.44	0.222
Ammonia Recycling	31	0.588	3	0.0186	1	0.288
Warburg Effect	57	1.08	4	0.0192	1	0.288
Phosphatidylethanolamine Biosynthesis	12	0.228	2	0.0201	1	0.288
Glycine and Serine Metabolism	59	1.12	4	0.0216	1	0.288
Amino Sugar Metabolism	33	0.626	3	0.022	1	0.288
Glucose-Alanine Cycle	13	0.247	2	0.0235	1	0.288
Purine Metabolism	73	1.38	4	0.0435	1	0.474
Glycolysis	23	0.436	2	0.068	1	0.595
Arginine and Proline Metabolism	52	0.986	3	0.0709	1	0.595
Inositol Phosphate Metabolism	24	0.455	2	0.0734	1	0.595
Glycerolipid Metabolism	25	0.474	2	0.0789	1	0.595
Phospholipid Biosynthesis	29	0.55	2	0.102	1	0.603
Inositol Metabolism	30	0.569	2	0.108	1	0.603
Starch and Sucrose Metabolism	31	0.588	2	0.114	1	0.603
Fructose and Mannose Degradation	31	0.588	2	0.114	1	0.603
Gluconeogenesis	33	0.626	2	0.127	1	0.603
Beta-Alanine Metabolism	34	0.645	2	0.133	1	0.603
Nicotinate and Nicotinamide Metabolism	35	0.664	2	0.14	1	0.603
Aspartate Metabolism	35	0.664	2	0.14	1	0.603
Phenylacetate Metabolism	9	0.171	1	0.159	1	0.603
Homocysteine Degradation	9	0.171	1	0.159	1	0.603
Lactose Degradation	9	0.171	1	0.159	1	0.603
De Novo Triacylglycerol Biosynthesis	9	0.171	1	0.159	1	0.603
Sphingolipid Metabolism	40	0.758	2	0.174	1	0.603
Malate-Aspartate Shuttle	10	0.19	1	0.175	1	0.603
D-Arginine and D-Ornithine Metabolism	11	0.209	1	0.191	1	0.603
Glycerol Phosphate Shuttle	11	0.209	1	0.191	1	0.603
Trehalose Degradation	11	0.209	1	0.191	1	0.603
Cardiolipin Biosynthesis	11	0.209	1	0.191	1	0.603
Glutamate Metabolism	48	0.91	2	0.23	1	0.702
Phosphatidylcholine Biosynthesis	14	0.265	1	0.236	1	0.702
Alpha Linolenic Acid and Linoleic Acid Metabolism	17	0.322	1	0.28	1	0.748
Alanine Metabolism	17	0.322	1	0.28	1	0.748
Spermidine and Spermine Biosynthesis	18	0.341	1	0.294	1	0.748
Pyrimidine Metabolism	57	1.08	2	0.295	1	0.748
Mitochondrial Electron Transport Chain	19	0.36	1	0.307	1	0.748
Lactose Synthesis	19	0.36	1	0.307	1	0.748
Nucleotide Sugars Metabolism	20	0.379	1	0.321	1	0.748
Catecholamine Biosynthesis	20	0.379	1	0.321	1	0.748
Glutathione Metabolism	20	0.379	1	0.321	1	0.748
Transfer of Acetyl Groups into Mitochondria	22	0.417	1	0.347	1	0.79
Tyrosine Metabolism	70	1.33	2	0.388	1	0.85
Cysteine Metabolism	26	0.493	1	0.396	1	0.85
Phenylalanine and Tyrosine Metabolism	27	0.512	1	0.408	1	0.85
Selenoamino Acid Metabolism	27	0.512	1	0.408	1	0.85
Pentose Phosphate Pathway	29	0.55	1	0.431	1	0.861
Folate Metabolism	29	0.55	1	0.431	1	0.861
Lysine Degradation	30	0.569	1	0.442	1	0.866
Androgen and Estrogen Metabolism	33	0.626	1	0.474	1	0.911
Propanoate Metabolism	42	0.796	1	0.56	1	1
Methionine Metabolism	42	0.796	1	0.56	1	1
Histidine Metabolism	42	0.796	1	0.56	1	1
Valine, Leucine and Isoleucine Degradation	59	1.12	1	0.688	1	1
Tryptophan Metabolism	59	1.12	1	0.688	1	1
Arachidonic Acid Metabolism	67	1.27	1	0.735	1	1
